# Recombinant immunotoxin induces tumor intrinsic STING signaling against head and neck squamous cell carcinoma

**DOI:** 10.1038/s41598-023-45797-7

**Published:** 2023-10-28

**Authors:** Guiqin Xie, Liang Shan, Cuicui Yang, Yuanyi Liu, Xiaowu Pang, Shaolei Teng, Tzyy-Choou Wu, Xinbin Gu

**Affiliations:** 1 Department of Oral Pathology, Howard University, 600 W Street NW, Washington, DC 20059 USA; 2 Cancer Center, Howard University, 2041 Georgia Avenue NW, Washington, DC 20059 USA; 3 Angimmune LLC, Rockville, MD 20855 USA; 4 Department of Biology, Howard University, 415 College St. NW, Washington, DC 20059 USA; 5 Pathology, Oncology, Obstetrics and Gynecology, and Molecular Microbiology and Immunology, Johns Hopkins University School of Medicine, Baltimore, MD 21287 USA

**Keywords:** Cancer therapy, Head and neck cancer, Tumour immunology

## Abstract

The innate immune stimulator of interferon genes (STING) pathway is known to activate type I interferons (IFN-I) and participate in generating antitumor immunity. We previously produced hDT806, a recombinant diphtheria immunotoxin, and demonstrated its efficacy against head and neck squamous cell carcinoma (HNSCC). However, it’s unknown whether the tumor-intrinsic STING plays a role in the anti-HNSCC effects of hDT806. In this study, we investigated the innate immune modulation of hDT806 on HNSCC. hDT806 significantly upregulated the level of STING and the ratio of p-TBK1/TBK1 in the HNSCC cells. Moreover, intratumoral hDT806 treatment increased the expression of STING-IFN-I signaling proteins including IFNA1, IFNB, CXCL10 and MX1, a marker of IFN-I receptor activity, in the HNSCC xenografts. Overexpression of STING mimicked the hDT806-induced upregulation of the STING-IFN-I signaling and induced apoptosis in the HNSCC cells. In the mouse xenograft models of HNSCC with STING overexpression, we observed a significant suppression of tumor growth and reduced tumor weight with increased apoptosis compared to their control xenograft counterparts without STING overexpression. Collectively, our data revealed that hDT806 may act as a stimulator of tumor-intrinsic STING-IFN-I signaling to inhibit tumor growth in HNSCC.

## Introduction

Head and neck squamous cell carcinoma (HNSCC) is an aggressive malignant disease, which accounts for about 90% of all diagnosed cases of head and neck cancers^1,2^. HNSCC patients are often diagnosed at an advanced loco-regional or metastatic stage of the disease, and experience significant morbidity associated with the traditional treatments, which range from surgery to chemo and radiotherapy^3,4^. Immunotherapy exploiting a patient’s own immune system to eliminate tumor cells has become one of the most prominent new cancer treatment options. Indeed, the recent FDA approval of immune checkpoint blockade (ICB) has altered the landscape of HNSCC therapy^5^ and achieved a durable clinical response in some patients. However, despite the promise ICB has brought to the clinics, this treatment is ineffective in over 80% of HNSCC patients, regardless of the human papillomavirus (HPV) status^6,7^. This highlights an unmet need for more treatment options that offer improved efficacy for patients with HNSCC. Recent studies have identified the innate immune stimulator of interferon genes (STING) pathway, a cytosolic DNA-sensing pathway that drives activation of type I interferons (IFN-I) and other inflammatory cytokines, in the generation of antitumor immune responses^8^. Pharmacological activation of STING in HNSCC cells was shown to enhance cell death through regulation of reactive oxygen species, beyond its canonical role in DNA damage sensing^9^. Analysis of tumors from HNSCC patient specimens revealed that low STING expression is associated with worse outcomes^9^. Indeed, STING agonists have been investigated in a completed clinical trial (NCT04144140) and an ongoing (NCT05070247) trial to treat patients with advanced solid tumors, including HNSCC. STING is a favorable prognosticator of HNSCC patients^10^, but STING is often inhibited in cancers. Moreover, it’s been identified that in immune-resistant HNSCC cells, the DNA-sensing defense response, mainly constituted of IFN-I signatures, is the most suppressed pathway^10^. The preclinical and clinical evidence underlines the critical importance of further exploration into tumor-intrinsic STING-IFN-I signaling as a potential treatment of HNSCC.

One of the most notable characteristics of HNSCC is that 90% of the tumors overexpress epidermal growth factor receptor (EGFR) to promote tumor growth. Cancer cells may further accumulate EGFR with gene amplification in 10–58%^11,12^ or a mutant EGFRvIII in up to 42% of tumors^13,14^, and establish a suppressive tumor microenvironment (TME)^15–17^, suggesting that EGFR may serve as an ideal target for drug development. However, cetuximab, as the only FDA-approved anti-EGFR antibody in the US, has shown quite disappointing efficacy as monotherapy in HNSCC with a 10–30% response rate^18–20^. Recombinant immunotoxin represents a promising therapeutic for cancer therapy, which is a fusion protein that combines a toxin, such as diphtheria toxin (DT), with an antibody or other targeting protein that binds specifically to a certain type of cell. Immunotoxin has been extensively investigated to target a tumor-specific antigen and directly kill tumor cells via protein synthesis suppression^21^. Immunotoxin has been approved by the FDA to treat certain types of hematopoietic malignancy since it produces complete regression and prolongs survival^22–27^. However, the application of immunotoxin in a solid tumor is largely underdeveloped.

In an effort to utilize EGFR overexpression in HNSCC as a drug development target, we previously engineered a bispecific and bivalent immunotoxin^28^, DT390-HuBiscFv806, abbreviated as hDT806, targeting the tumor-specific overexpressed EGFR and/or EGFRvIII mutant. We have demonstrated that hDT806 was highly effective in eliminating tumors or suppressing tumor growth in preclinical settings of HNSCC as well as glioblastoma^28,29^. In HNSCC, we showed that hDT806 effectively suppressed cell proliferation in the HNSCC lines tested, distinct from the EGFR-targeted tyrosine kinase inhibitor erlotinib or the antibody cetuximab. We further demonstrated that the direct disruption of EGFR signaling, transcription inhibition, DNA damage as well as apoptosis induced by hDT806 may contribute to its antitumor efficacy in HNSCC^29^. However, whether the tumor-intrinsic STING plays a role in the anti-HNSCC effects of hDT806 is unknown. In the current study, we set out to investigate the role of tumor-intrinsic STING in mediating hDT806’s cytotoxicity against HNSCC. We found that hDT806 treatment activated the tumor-intrinsic STING-IFN-I signaling pathway, which in turn inhibited tumor growth by inducing apoptosis in HNSCC. Our data demonstrate the significance of the tumor-intrinsic STING-IFN-I signaling axis in the hDT806-induced tumor growth inhibition, and provide valuable insights into the development of novel immunotherapeutic approaches for treating HNSCC.

## Materials and methods

### Cell culture

The JHU-029 and JHU-013 HNSCC cell lines derived from patient HNSCC were a kind gift from the Johns Hopkins University (Baltimore, MD, USA)^28–30^. The JHU-013, JHU-029, JHU-029 with control vector (designated as JHU029-control) and JHU-029 with STING overexpression (designated as JHU029-STING) were cultured in RPMI-1640. The 293FT cells were from Thermo Fisher Scientific Inc. (#R70007; Waltham, MA USA) and maintained in DMEM. The culture media were supplemented with 10% fetal bovine serum (FBS) and 1% antibiotic–antimycotic solution. All the cells were grown in a humidified incubator at 37 °C in 5% CO_2_.

### Plasmids, transfection, and viral transduction

The STING expression plasmid (#102586) and lentiviral packaging vector plasmids, pCMV-dR8.2 dvpr (#8455) and pCMV-VSV-G (#8454), were obtained from Addgene (Watertown, MA, USA). pLVX-IRES-Puro, which does not have ectopic STING overexpression, was obtained from Clontech Laboratories, Inc (Mountain View, CA, USA) and used as the control vector plasmid. To generate control and STING-expressing lentivirus for the establishment of control or STING-expressing cells, lentiviral packaging was conducted in 293FT cells. Co-transfection of either the control or STING-expressing plasmid, pCMV-dR8.2 dvp, and pCMV-VSV-G plasmids was performed. The 293FT cells were plated and allowed to reach approximately 80% confluence the following day for transfections. Lipofectamine 2000 (Cat. No. 11668019, Thermo Fisher Scientific) was used for the co-transfection of the three plasmids in the 293FT cells, following the manufacturer's instructions. After 48–72 h post-transfection, the supernatant from the 293FT cells was collected and concentrated for viral transduction. To establish the control and STING-overexpressing HNSCC lines, JHU-029 cells were plated 24 h prior to viral transduction. On the day of transduction, when the cells reached approximately 50% confluence, viral transduction was performed in a 6-well plate, as we previously described^31^. Briefly, the viruses were incubated with cells in Opti-MEM™ medium (Cat. No. 31985062, Thermo Fisher Scientific) in the presence of 10 μg/mL polybrene (Cat. No. TR-1003-G; Sigma-Aldrich, St. Louis, MO, USA) at 37°C for 5 h. The transduced cells were replenished with complete RPMI-1640 media for two days. Then, the transduced cells were selected with 2.5 μg/mL puromycin for 4 days.

### Reverse transcriptase PCR (RT-PCR)

To isolate total RNA from JHU-029 and -013 cells, human peripheral blood mononuclear cells (PBMCs; Sylvan N. Goldman Oklahoma Blood Institute, Oklahoma, USA), and Jurkat cells (Clone E6-1, ATCC, VA, USA), an RNeasy Mini Kit (#74106, Qiagen, Germantown, MD, USA) was used following the manufacturer’s instruction. RNA concentrations were measured on a NanoDrop 2000c spectrophotometer (ND-2000c, Thermo Scientific, Wilmington, DE, USA) and RNA samples were stored at − 80 °C. RT-PCR was performed in a total volume of 12 µL using 6 µL of 2 × Luna Universal One-Step Reaction Mix (#E3005, New England Biolabs, MA, USA), 0.6 µL of RT Enzyme Mix (20 ×), 1 µL of 5 µM primer for each primer per reaction, 2 µL of the RNA dilution (100 ng/mL), and 2.4 µL water. The following primers were used: (1) For the *EGFR* gene: Forward, 5′-CCA GTA TTG ATC GGG AGA GC-3′; reverse, 5′-CCA AGG ACC ACC TCA CAG TT-3′. (2) For the *GAPDH* gene: Forward, 5′-GGGAAGGTGAAGGTCGGAGT-3′; reverse, 5′-GGAGGGATCTCGCTCCTG-3′. The PCR cycling on a PCR thermocycler (Applied Biosystems, Life Technologies, CA, USA) was performed as follows: a reverse transcription step (55 °C, 10 min) and an initial denaturation step (95 °C, 1min), followed by 40 cycles of denaturation (95 °C, 10 s), extension (60 °C, 60 s). Gel electrophoresis was performed for the reactions. The fold-change for *EGFR* gene expression relative to that of *GAPDH* at baseline was determined and analyzed using the ImageJ software.

### Western blot analysis

Western blot analysis was performed as previously described^29,31^. Briefly, cells seeded in the wells of a six-well plate were treated with either vehicle or hDT806 (20 nM) for 48 h before they were collected, washed with PBS, and homogenized in RIPA lysis buffer (50 mM Tris buffer, 150 mM NaCl, 1 mM EDTA, 1% NP40, 0.5% (w/v) Sodium Deoxycholate, 0.1% (w/v) SDS and proteinase/phosphatase inhibitors). After homogenizing the cells, they were subjected to centrifugation at 16,000×*g* for 20 min at 4 °C. The resulting lysates were then transferred to a new Eppendorf tube. The protein concentrations of these lysates were measured, and lysates (30 μg/lane) were utilized for the Western blot analysis. The antibodies against p-p38 MAPK (#9216), p38 MAPK (#9212), p-NF-kB p65 (#3033), NF-kB p65 (#6956), poly (ADP ribose) polymerase 1 (PARP) (#9542), p-TBK1 (#5483), TBK1 (#3504), and STING (#13,647) were purchased from Cell Signaling (Beverly, MA, USA). Antibodies against CXCL10 (#10937-1-AP), IFNA1 (#18013-1-AP), IFNB (#27506-1-AP), MX1 (#13750-1-AP) were purchased from Proteintech (Rosemont, IL, USA) and MYC (ab32072), PD-L1 (ab210931) from Abcam (Cambridge, MA, USA). Antibodies against β-actin (#47778 HRP), SOX2 (sc-365823 HRP), and ALDH1/2 (sc-166362 HRP) were purchased from Santa Cruz (Dallas, TX, USA). Antibody against caspase-9 (#c7729) was from Sigma-Aldrich. Anti-rabbit or anti–mouse IgG conjugated with Horseradish peroxidase was used as the secondary antibody where appropriate. The primary antibodies were used at a 1:1000 dilution and the secondary antibodies were used at a 1:2000 dilution following the manufacturers’ instructions. We utilized an ECL system to identify protein bands, and then quantified the intensity of these bands using the ImageJ software.

### Immunohistochemical (IHC) analysis

Immunohistochemistry was performed as previously described^29,31^, on formalin-fixed paraffin-embedded tumor sections (5 μm) using primary antibodies at a 1:50–500 dilution following the manufacturers’ instructions directed against STING (#13647; Cell signaling), Ki-67 (#RB-9043-P0; Thermo Fisher Scientific), cleaved caspase-3 (#9664; Cell Signaling), IFNA1 (#18013-1-AP, Proteintech), IFNB (#27506-1-AP, Proteintech), CXCL10 (#10937-1-AP, Proteintech), and MX1 (#13750-1-AP, Proteintech). The staining was achieved by incubating with diaminobenzidine (DAB) for 5 min using a DAB peroxidase substrate kit (Vector Laboratories, Burlingame, CA, USA). IHC analysis was performed in triplicates for this study. On each slide, cell counting was conducted at 40X magnification, focusing on five randomly selected fields with a minimum count of 500 cells. Cells showing negative or positive staining by antibodies were counted separately. The percentage of cells exhibiting positive staining was calculated using the following formula: [(number of positively stained cells divided by the sum of negatively and positively stained cells) × 100%].

### Flow cytometry apoptosis assay

Apoptosis flow cytometry assay was performed as previously described^29,31^. Briefly, cells were treated with either vehicle or hDT806 (20 nM) for 48 h, then collected and incubated with annexin V‐FITC and propidium iodide (PI) solutions in the dark for 15 min. Flow cytometry analysis was conducted using a BD flow cytometer (BD Bioscience, San Jose, CA, USA), and apoptotic cells were identified as annexin V-positive and PI-negative, or annexin V-positive and PI-positive cells. The percentage of apoptotic cells was determined using FlowJo software (FlowJo LLC, Ashland, OR, USA).

### Xenograft tumor model

We previously established the JHU-029 xenograft tumor model as described^29^. Briefly, we utilized 6–8-week-old NOD scid gamma mouse (NSG) mice. A volume of 200 μL serum-free medium containing 45% Matrigel basement membrane matrix (Cat. 354234; BD Biosciences, Billerica, MA) and five million cells were subcutaneously inoculated into the right flank of mice 2–3 weeks prior to treatment. Treatment with either vehicle or hDT806 began when the median tumor size reached approximately 80 mm^3^. We administered hDT806 (12 μg/kg/mouse) via intratumoral injection every other day. We measured the tumor size using a caliper and body weight of the mice once every 2–3 days. After 26 days of treatment, we euthanized the mice by cervical dislocation under 4% isoflurane anesthesia and dissected the tumors for downstream IHC analysis.

To establish the JHU029-control and JHU029-STING xenograft tumor models without and with STING overexpression, respectively, in 6–8-week-old NSG mice, we inoculated the JHU029-control and JHU029-STING cells (5 million cells in 200 μL serum-free medium containing 45% Matrigel basement membrane matrix) on opposite flanks of the same mice to mitigate any confounding factors. We measured the tumor size using a caliper and body weight of the mice once every 2–3 days. At the end of the experiment on day 35, we euthanized the mice by cervical dislocation under 4% isoflurane anesthesia and dissected the tumors for downstream analysis.

### Statistical analysis

The results are presented as mean ± standard error of mean (SEM). Statistical analysis was performed using the Student t-test, paired t-test, and one-way analysis of variance (ANOVA) as appropriate. All tests were two-sided, and *p*-values less than 0.05 were considered statistically significant.

### Compliance with ethical standards

All animal experiments were performed under protocols approved by the Howard University Animal Care and Use Committee (IACUC-MED-17-01). The methods employed in the research were in full compliance with the relevant guidelines and regulations governing the ethical treatment and use of animals in scientific research. The study is reported in accordance with ARRIVE guidelines (https://arriveguidelines.org).

## Results

### hDT806 activates STING-IFN-I axis in the HNSCC cells

**Figure 1:**
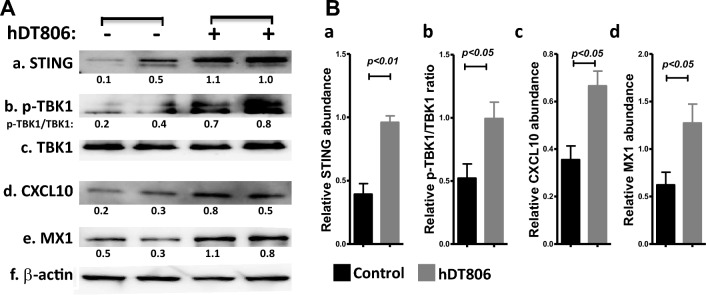
hDT806 stimulates STING-IFN-I signaling in HNSCC cells. (**A**) Total protein extracts were prepared from the cells treated with vehicle or hDT806 (20 nM). Western blot analysis was performed for STING (a), p-TBK1 (b), TBK1 (c), CXCL10 (d), MX1 (e), and β-actin (f) in the JHU-029 HNSCC cells treated with vehicle or hDT806 for 48 h. (**B**) Protein band intensities of STING (a), CXCL10 (c), and MX1 (d) relative to the corresponding β-actin, and the ratio of p-TBK1 /TBK1 (b) were quantified for comparisons between the vehicle-treated cells and the hDT806-treated cells. Data of three or four independent experiments are presented as mean ± SEM, with *p* values indicated.

It’s known that 90% HNSCC contains EGFR overexpression^32,33^. hDT806 was engineered to target tumor-specific overexpressed EGFR and/or EGFRvIII mutants. We recently reported that strong EGFR protein and gene expression were observed in HNSCC cells, which were significantly reduced by hDT806 treatment^29^. To directly assess the level of *EGFR* gene expression in comparison to other tissues, we performed an RT-PCR experiment using Jurkat cells, human PBMCs, JHU-013, and JHU-029 cells, respectively. Compared to the weak expression of *EGFR* in Jurkat cells and PBMCs, both JHU-013 and JHU-029 cells exhibited much stronger *EGFR* expression (Supplementary Fig. S1). Our recent work in HNSCC showed that hDT806 can induce a strong increase in rH2A.X^29^, which represents an early cellular response to DNA double-strand breaks and a marker of DNA damage^34^. Poly (ADP-ribose) polymerase (PARP) is a DNA-binding protein which is primarily activated by nicks in the DNA molecule. We also reported^29^ that hDT806 caused a significant increase in the cleaved PARP, a well-established apoptotic cell death marker known to be associated with DNA damage response^35^. Here, we hypothesized that the hDT806-induced DNA damage may activate the cyclic guanosine monophosphate–adenosine monophosphate (cyclic GMP-AMP) synthase (cGAS)/STING pathway, which is known to be triggered by cytosolic DNA, either exogenous or endogenous, and has been established as a critical activator of anti-tumor immune responses^36^. In our previous study, we found that JHU-029 cells were more sensitive to hDT806 than small molecular inhibitors compared to a range of HNSCC cell lines^29^. We first examined whether hDT806 could affect STING protein expression levels in the JHU-029 cells by western blotting. The level of STING protein in JHU-029 with hDT806 treatment was significantly elevated to 2.4 folds (n = 4, *p* < 0.05; Fig. 1Aa,Ba), compared to the vehicle treatment. Since intracellular STING elevation can activate downstream transcription factors through TBK1 to stimulate the IFN-I response^37^, we then assessed if hDT806 stimulates downstream effectors of STING signaling in HNSCC cells. We found that the phosphorylation of TBK1 increased while the level of total TBK1 didn’t change, which elevated the ratio of p-TBK1/TBK to 1.8 folds (n = 4, *p* < 0.05; Fig. 1Ab-c,Bb) in the hDT806 treatment vs vehicle treatment group. Chemokine (C-X-C motif) ligand 10 (CXCL10), a chemokine related to T cell recruitment, also known as IFN-γ–inducible protein 10 (IP-10), is a well-established downstream effector of the IFN-I signaling^37^. Indeed, we found that the level of CXCL10 was increased to 1.9 folds (n = 3, *p* < 0.05; Fig. 1Ad,Bc) in the hDT806 treatment vs vehicle treatment group. Consistently, MX dynamin-like GTPase 1 (MX1)^38^, an interferon-induced GTP-binding protein, which is known to be one of the cardinal IFN-I-stimulated gene products as well as a sensitive indicator of IFN-I receptor (IFNAR) signaling, was increased to 2.0 folds (n = 4, *p* < 0.05; Fig. 1Ae,Bd) in the hDT806 treatment group compared to that of the vehicle treatment group. To determine whether the response of STING-IFN-I signaling to hDT806 is unique to JHU-029 cells, we analyzed changes in key proteins within this pathway after hDT806 treatment in another HNSCC line, JHU-013. Indeed, JHU-013 cells exhibited similar changes, with significantly increased levels of STING and MX1, as well as an elevated ratio of p-TBK1/TBK1 in response to hDT806 treatment (Supplementary Fig. S2). These findings suggest that hDT806 activation of the STING signaling pathway is not specific to JHU-029 cells and is also observed in JHU-013 cells, indicating its potential broader applicability in HNSCC cell lines.

Taken together, these data suggest that hDT806 treatment may activate tumor cell-intrinsic STING-IFN-I axis in HNSCC.

### hDT806 stimulates STING-IFN-I axis in the HNSCC xenograft tumors

**Figure 2:**
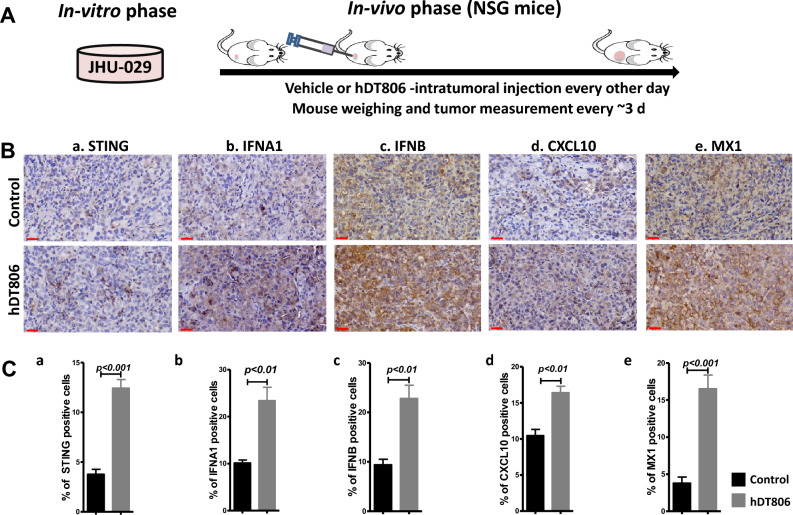
hDT806 treatment enhances STING-IFN-I axis activities in HNSCC xenografts. (**A**) A schema shows the treatment procedure previously reported^29^. When the JHU-029 HNSCC xenograft tumors reached an average size of ~ 80 mm^3^, intratumoral injection of vehicle or hDT806 was administered for 26 days. (**B**) The dissected tumors from NSG mice treated with vehicle or hDT806 after 26-day treatment were subject to immunohistochemistry (IHC) analysis for STING (a), IFNA1 (b), IFNB (c), CXCL10 (d), and MX1 (e). Inset scale bars = 30 µm. (**C**) Quantification of the IHC analysis shows hDT806 treatment increases the expression of STING (a), IFNA1 (b), IFNB (c), CXCL10 (d), and MX1 (e) in the JHU-029 xenograft tumors, compared to vehicle treatment. Data are presented as mean ± SEM (n = 4), with *p* values indicated.

Since hDT806 treatment significantly activated tumor cell-intrinsic STING-IFN-I axis in HNSCC, we proceeded to assess whether the effects of hDT806 in activating the tumor-intrinsic STING-IFN-I axis could be recapitulated in vivo. We previously established JHU-029 HNSCC xenograft tumor models in NSG mice^29^: in these models, when the tumors reached an average size of ~ 80 mm^3^, intratumoral administration of vehicle or hDT806 was administered (12 μg/kg every other day) for 26 days (Fig. 2A). We reported that intra-tumoral injection of hDT806 significantly suppressed tumor growth and reduced tumor weight compared to vehicle treatment. The in vivo data support the notion that hDT806 effectively inhibited the growth of HNSCC tumors in mice^29^. We performed IHC analysis on the STING, type I IFNs (including IFNA1 and IFNB), CXCL10, and MX1 proteins of the dissected tumors from NSG mice after 26 days of vehicle or hDT806 treatment. IHC experiment revealed a dramatic increase in STING-positive cells from 3.8 ± 0.5 to 12.4 ± 0.9% (n = 4, *p* < 0.001; Fig. 2Ba,Ca), IFNA1-positive cells from 10.2 ± 0.6 to 23.4 ± 2.8% (n = 4, *p* < 0.01; Fig. 2Bb,Cb), IFNB-positive cells from 9.4 ± 1.1 to 22.8 ± 2.7% (n = 4, *p* < 0.01; Fig. 2Bc,Cc), CXCL10-positive cells from 10.5 ± 0.9 to 16.4 ± 0.9% (n = 4, *p* < 0.01; Fig. 2Bd,Cd), and MX1-positive cells from 3.8 ± 0.8 to 16.5 ± 1.9% (n = 4, *p* < 0.001; Fig. 2Be,Ce) in the hDT806-treated, compared to the vehicle-treated tumors, respectively.

Additionally, we evaluated the expression of STING in the GL261vIII mouse glioblastoma models with overexpression of EGFRvIII mutant, which shows a particular sensitivity to hDT806 in our previous studies^28^. In line with the effects of hDT806 in HNSCC xenograft models, indeed, an IHC experiment of the dissected GL261vIII tumors revealed a dramatic increase in STING-positive cells in hDT806-treated tumors compared to the vehicle-treated tumors, from 1.1 ± 0.4 to 9.9 ± 0.5% (n = 4, *p* < 0.001; Supplementary Fig. S3), indicating that STING upregulation occurs in response to hDT806 in both glioblastoma and HNSCC.

Taken together, our results indicate that intratumoral hDT806 treatment activates the tumor-intrinsic STING-IFN-I axis in HNSCC in vivo.

### hDT806 elevates the activities of p38 and NF-ĸB p65 by phosphorylation in the HNSCC cells

**Figure 3:**
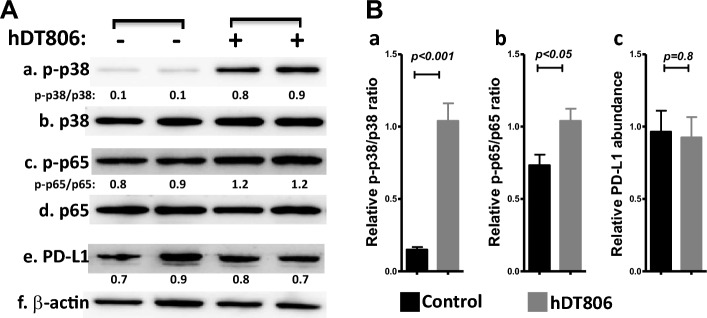
hDT806 stimulates p38 MAPK and NF-kB p65 in HNSCC cells by phosphorylation. (**A**) Total protein extracts were prepared from the cells treated with vehicle or hDT806 (20 nM). Western blot analysis was performed for p-p38 (a), p38 (b), p-p65 (c), p65 (d), PD-L1 (e), and β-actin (f) in the cells treated with vehicle or hDT806 for 48 h. (**B**) Protein band intensities of the ratios of p-p38/p38 (a) and p-p65/p65 (b), and PD-L1 (c) relative to the corresponding β-actin were quantified for comparisons between the vehicle-treated cells and the hDT806-treated cells. Data of three or four independent experiments are presented as mean ± SEM, with *p* values indicated.

We recently found^29^ that immunotoxin hDT806 increases phosphorylation of extracellular signal-regulated kinase 1/2 (ERK1/2), a member of the mitogen-activated protein kinases (MAPKs). It was previously reported that a pseudomonas exotoxin (PE)-based immunotoxin stimulates phosphorylation of p38 MAPK^39^, a kinase that can modulate the nuclear factor (NF)-κB p65^40^. The inflammatory protein NF-ĸB is a key regulatory protein that can initiate cell death^41,42^. Since STING activation is known to trigger several inflammatory pathways including NF-κB, we assessed whether hDT806 could affect p38 and p65 proteins in HNSCC. After treating HNSCC cells with hDT806 for 48 h, compared to the vehicle treatment group, we found that hDT806 treatment enhanced the phosphorylation of p38 while it exhibited minimal effect on the total p38 protein. Similarly, increased NF-κB p65 phosphorylation by hDT806 was also observed while the total NF-κB p65 protein was not altered. Specifically, the hDT806 treatment elevated the ratio of p-p38/p38 to 7.6 folds (n = 4, *p* < 0.05; Fig. 3Aa-b,Ba), and p-p65/p65 to 1.4 folds (n = 4, *p* < 0.05; Fig. 3Ac-d,Bb), respectively, compared to those from the vehicle treatment group.

Since hDT806 treatment upregulated NF-κB p65 activity and NF-κB as a master transcription factor of inflammation and immunity is emerging as a key positive regulator of programmed death ligand 1 (PDL1) expression in cancer^43^, we assessed whether this effect of hDT806 on p65 would result in any changes in PDL1, one of the most extensively studied inhibitory immune checkpoints due to its critical role in cancer immunotherapy. In contrast to its effects on p-p38 or p-p65, western blot analysis showed that hDT806 treatment only altered PDL1 to 0.96 folds (n = 3, *p* > 0.05; Fig. 3Ae,Bc), compared to its counterpart vehicle treatment. Shin et al. recently reported^44^ that NF-κB activity alone may not be sufficient to induce PDL1 expression; instead, PDL1 expression might increase following treatment with IFN-γ in HNSCC. Taken together, these data suggest that while hDT806 enhances not only p38 MAPK but also NF-ĸB p65 activity by phosphorylation, and these activities do not necessarily lead to an alteration in the inhibitory immune checkpoint PDL1 in HNSCC cells with the current experimental conditions.

### hDT806 decreases the protein levels of SOX2 and MYC in the HNSCC cells

**Figure 4:**
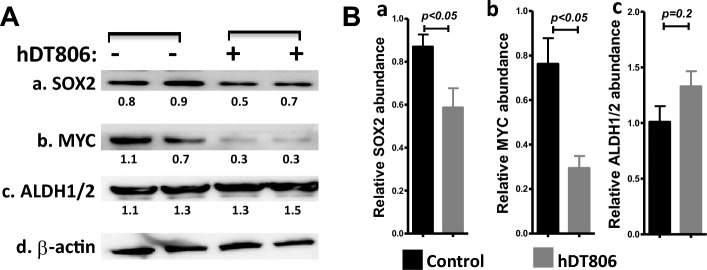
hDT806 decreases SOX2 and MYC in HNSCC cells. (**A**) Total protein extracts were prepared from the cells treated with vehicle or hDT806 (20 nM). Western blot analysis was performed for SOX2 (a), MYC (b), ALDH1/2 (c), and β-actin (d) in the cells treated with vehicle or hDT806 for 48 h. (**B**) Band intensities of SOX2 (a), MYC (b), and ALDH1/2 (c) relative to the corresponding β-actin were quantified for comparisons between the vehicle-treated cells and the hDT806-treated cells. Data of three or four independent experiments are presented as mean ± SEM, with *p* values indicated.

Recent studies indicated that sex-determining region Y [SRY]-box (SOX)2, a key transcription factor involved in maintaining the pluripotency of cancer stem cells (CSCs), was aberrantly expressed in HNSCC^45^. CSCs are known to be responsible for drug resistance and cancer relapse^46^. It was shown that SOX2 may have important roles in the ‘stemness' and progression of HNSCC^47^. SOX2 has been reported to dampen the immunogenicity of HNSCC by targeting the STING pathway for degradation^48^. Therefore, to evaluate if the hDT806-induced STING is associated with an alteration of SOX2 in HNSCC, we assessed SOX2 expression levels in response to hDT806 treatment. Indeed, we found that 48 h treatment of hDT806 significantly reduced SOX2 protein levels to 67.5% (n = 4, *p* < 0.05; Fig. 4Aa,Ba) in the JHU-029 cells, compared to the vehicle treatment.

MYC is a proto-oncogene with crucial role in tumor initiation, progression, and maintenance. The MYC-induced oncogenic and epigenetic reprogramming leads to the acquisition of cancer stem-like cell-associated properties and the induction of intratumoral heterogeneity^49^. MYC was recently reported to promote immune-suppression in triple-negative breast cancer via inhibition of STING-IFN-I signaling in a tumor cell-intrinsic fashion via direct transcriptional repression^50^. Consistent with the downregulation of SOX2 protein levels, hDT806 also affected the MYC oncoprotein in JHU-029, decreasing the level of MYC expression to 38.7% (n = 4, *p* < 0.05; Fig. 4Ab,Bb) compared to the vehicle treatment group.

We additionally evaluated aldehyde dehydrogenase 1/2 (ALDH1/2), another useful marker for identification of CSCs in HNSCC^51^. Western blot analysis did not show significant changes in the protein level of ALDH1/2 after hDT806 treatment (n = 3, *p* > 0.05; Fig. 4Ac,Bc), compared to the vehicle treatment group. Together, these data suggest that hDT806 treatment could differentially affect the proteins SOX2, MYC, and ALDH1/2 in HNSCC.

### Overexpression of STING resembles the hDT806-induced increase in STING-IFN-I axis in the HNSCC cells

**Figure 5:**
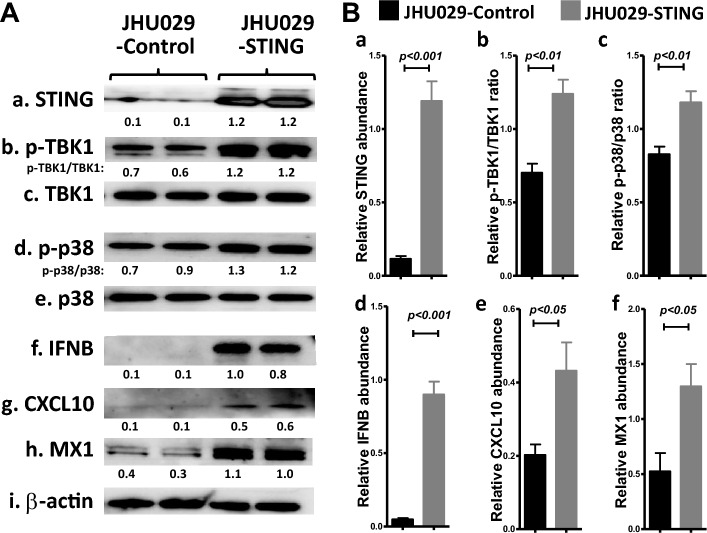
Overexpression of STING mimics hDT806’s stimulation of the STING-IFN-I axis in HNSCC cells. (**A**) Total protein extracts were prepared from the JHU-029 cells with (designated as JHU029-STING) or without (designated as JHU029-control) STING overexpression. Western blot analysis was performed for STING (a), p-TBK1 (b), TBK1 (c), p-p38 (d), p38 (e), IFNB (f), CXCL10 (g), MX1 (h), and β-actin (i). (**B**) Band intensities of STING (a), IFNB (d), CXCL10 (e), and MX1 (f) relative to the corresponding β-actin, the ratios of p-TBK1/TBK1 (b) and p-p38/p38 (c) were quantified for comparisons between the JHU029-STING and JHU029-control groups. Data of three or four independent experiments are presented as mean ± SEM, with *p* values indicated.

Since hDT806 treatment increased the proteins of the STING-IFN-I axis in the JHU-029 HNSCC cells as well as xenograft tumors, we overexpressed ectopic STING protein in JHU-029 to confirm that upregulated STING expression indeed leads to the activation of IFN-I in the HNSCC cells. Western blot analysis showed that ectopic STING overexpression elevated the level of STING to 10.4 folds (n = 4, *p* < 0.05; Fig. 5Aa,Ba), the ratio of p-TBK1/TBK to 1.7 folds (n = 4, *p* < 0.05; Fig. 5Ab-c,Bb) and p-p38/p38 to 1.4 folds (n = 4, *p* < 0.05; Fig. 5Ad–e,Bc), respectively; the level of IFNB to 20.7 folds (n = 4, *p* < 0.05; Fig. 5Af,Bd), CXCL10 to 2.1 folds (n = 4, *p* < 0.05; Fig. 5Ag,Be), and MX1 to 2.5 folds (n = 3, *p* < 0.05; Fig. 5Ah,Bf), respectively, compared to those in the JHU029-control cells. This set of data indicates that STING overexpression resembles the hDT806-induced upregulation of STING-IFN-I axis in the HNSCC cells.

### Overexpression of STING renders the HNSCC cells prone to apoptotic cell death

**Figure 6:**
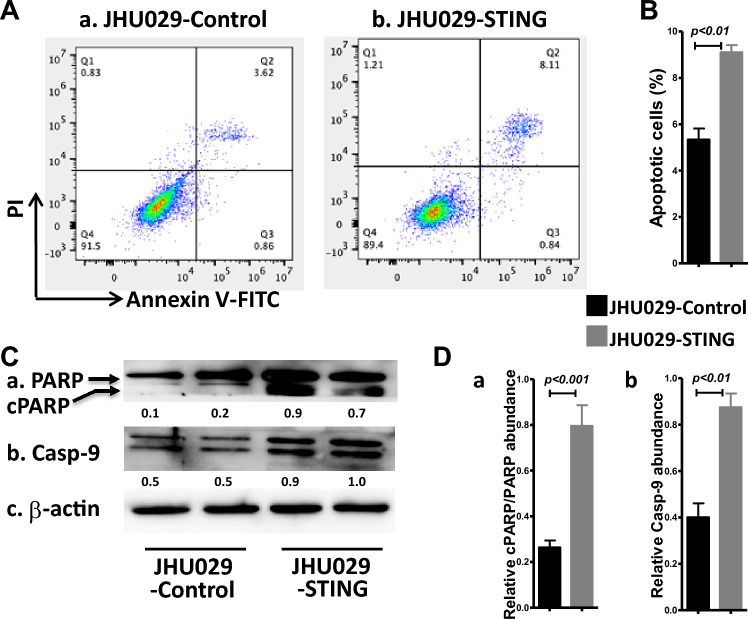
Overexpression of STING renders susceptibility to cell death of HNSCC cells. (**A**) Cells were collected for Annexin V and PI staining followed by flow cytometric analysis for apoptotic cells in the JHU-029 cells without (a) or with (b) STING overexpression. (**B**) The populations of apoptotic cells were quantified for the JHU-029 HNSCC cells with or without STING overexpression. Data of three independent experiments are presented as mean ± SEM (n = 3; *p* < 0.05). (**C**) Total protein extracts were prepared from the HNSCC cells with or without STING overexpression. Western blot analysis was performed for PARP and cleaved PARP (cPARP) (a), Caspase-9 (b), and β-actin (c). (**D**) The ratio of cleaved PARP/PARP (a) and band intensities of caspase-9 (b) relative to the corresponding β-actin were quantified for comparisons. Data of four independent experiments are presented as mean ± SEM, with *p* values indicated.

It was shown in B cell malignancy^52^ that STING activation and aggregation can directly trigger cancer cell death. A recent elegant research in HNSCC showed that STING serves as a tumor-intrinsic regulator of tumor cell survival and enhances cell death through regulation of reactive oxygen species and DNA damage^9^. We reported that JHU-029 showed more apoptotic cell death in response to hDT806 treatment^29^. Here, we found that the ectopically overexpressed STING on its own was capable of resulting in more cell apoptosis in JHU-029 compared to the control cells. Flow cytometry analysis revealed the apoptotic cells were significantly increased from 5.3% in the JHU029-control cells (Fig. 6Aa,B) to 9.1% in the JHU029-STING cells (Fig. 6Ab,B), indicating that STING overexpression per se could result in more apoptotic cell death.

To confirm the increased apoptotic events detected by flow cytometric analysis, we performed western blot analysis to evaluate several proapoptotic proteins, including cleaved PAPR and caspase-9, in JHU-029 with or without STING overexpression. Compared to the JHU029-control cells, we observed increased levels of proapoptotic proteins in the JHU029-STING cells. Specifically, the ratio of cleaved PARP relative to PARP (cPARP/PARP) showed a fold increase of 3.0 (n = 4, *p* < 0.001; Fig. 6Ca,Da), and the level of caspase-9 increased to 2.2 folds (n = 4, *p* < 0.01; Fig. 6Cb,Db). These findings demonstrate that STING overexpression in the cells promotes apoptotic cell death. Western blot analysis corroborated the flow cytometry results, further confirming the susceptibility of cells with STING overexpression to apoptosis.

### Overexpression of STING reduces tumor growth and increase apoptosis of the HNSCC xenograft tumors in NSG mice

**Figure 7:**
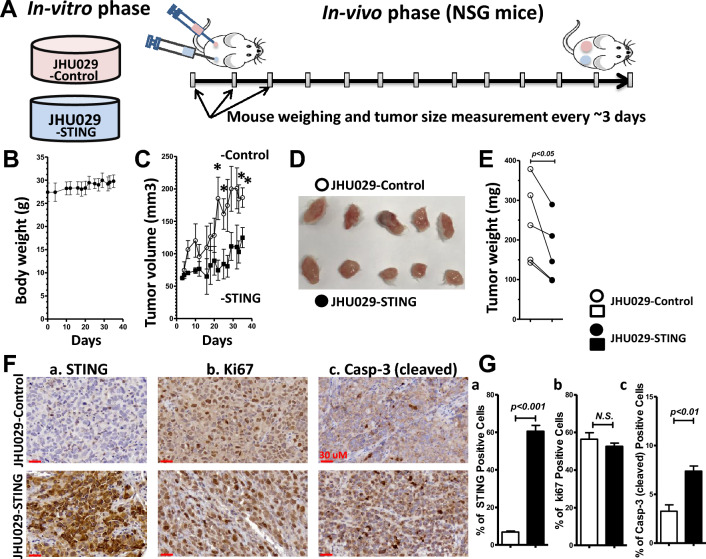
Overexpression of STING in HNSCC cells delays the xenograft tumor growth in NSG mice. (**A**) A schema represents the development of the HNSCC xenograft tumors with and without STING overexpression inoculated on the opposite flanks of same NSG mice for 35-day. (**B**) Average body weight of the NSG mice during the tumor development. (**C**) Tumor growth curves plotted for the HNSCC xenograft tumors with or without STING overexpression. *p < 0.05. (**D**) The JHU-029 HNSCC xenograft tumors with (lower panel) and without (upper panel) STING overexpression were harvested from the NSG mice. (**E**) Comparison of the average weight of the dissected tumors from NSG mice (n = 5; *p* < 0.05). (**F**) IHC images of STING (a), Ki67 (b) and cleaved caspase-3 (c) of the HNSCC xenograft tumors with or without STING overexpression. Inset scale bars = 30 µm. (**G**) Quantification of the IHC images reveals a dramatic increase in the expression of STING (a), minimal changes of Ki67 expression (b), and a significant increase in cleaved caspase-3 (c) expression in the JHU-029 HNSCC xenograft tumors with versus without STING overexpression. Data are presented as mean ± SEM (n = 4), with *p* values indicated.

To understand the impact of increased STING protein on HNSCC tumor growth, we investigated the effect of STING overexpression on xenograft tumor development in NSG mice. The JHU-029 cells with or without STING overexpression were inoculated on opposite flanks of the same NSG mice and tumor development was observed (Fig. 7A). Throughout the xenograft tumor development, the mice did not experience any reduction in body weight (Fig. 7B). While the tumor volume of both JHU-029 tumors with or without STING overexpression continuously increased, STING overexpression decelerated the xenograft tumor development. This effect was supported by the evidence that the tumor growth (Fig. 7C) as well as tumor weight in the JHU029-STING tumors were significantly downregulated, compared to the JHU029-control tumors (244.3 ± 45.8 vs 168.5 ± 36.8 mg; n = 5, *p* < 0.05 by paired student t-test; Fig. 7D,E). The in vivo data supported the notion that tumor-intrinsic STING overexpression effectively inhibits the growth of HNSCC tumors in mice.

To explore the mechanisms underlying the STING-induced inhibition of tumor growth, we examined cancer cell proliferation in the xenograft tumors. The STING overexpression and its effect on tumor growth were analyzed by IHC analysis of the xenograft tumor tissues. We measured STING and the crucial proteins for cell proliferation as well as apoptosis, Ki67 and cleaved caspase-3, respectively. STING overexpression in JHU-029 dramatically increased STING-positive cells, while the JHU-029 tumors without STING overexpression showed much less STING-positive cells (6.8 ± 0.5% vs. 60.6 ± 3.0%, n = 4, *p* < 0.001; Fig. 7Fa,Ga). Numerous Ki67-postive cells were found in the tissues of JHU-029 tumor with or without STING overexpression. The Ki67-postive cells were 56.4 ± 3.5% in the JHU029-control tumors to 52.6 ± 1.7% in the JHU029-STING tumors (n = 4, *p* > 0.05; Fig. 7Fb,Gb). On the contrary, the number of cleaved caspase-3-positive cells was significantly increased from 3.3 ± 0.6% in the JHU029-control tumors to 7.4 ± 0.5% in the JHU029-STING tumors (n = 4, *p* < 0.01; Fig. 7Fc,Gc). These findings are consistent with our in vitro experiments and support the notion that STING overexpression in JHU-029 induces apoptosis while having a minimal effect on proliferation in vivo.

## Discussion

We previously generated an immunotoxin, hDT806, with dual specificity by fusing an engineered DT fragment, DT390, with two single chain variable fragments of mAb806 targeting cancer-specific overexpressed EGFR and/or EGFRvIII mutant, and demonstrated the anti-tumor efficacy of hDT806 in glioblastoma^28^ as well as HNSCC^29^. Here, we further discovered a role of tumor-intrinsic STING/IFN-I signaling elicited by hDT806 is involved in hDT806’s cytotoxicity in HNSCC. We demonstrated that the cancer-specific EGFR targeted immunotoxin, hDT806, was able to act as a stimulator of the tumor-intrinsic innate immune response by activating the STING-IFN-I axis in both HNSCC cells as well as xenograft tumor models. Treatment with hDT806 also elevated the activities of p38 and NF-κB p65 by phosphorylation while decreased the protein levels of SOX2 and MYC in HNSCC cells. STING overexpression resembled the hDT806-induced increase in STING-IFN-I axis, rendered sensitivity to apoptotic cell death in HNSCC cells, promoted apoptosis and decelerated xenograft tumor growth in HNSCC mouse models.

Our current results revealed a role of tumor-intrinsic innate immune response, specifically the protein effectors of the STING-IFN-I signaling stimulated by hDT806 in tumor control in HNSCC. It’s known that the recognition of cytosolic DNA, whether it’s exogenous from pathogen invasion or endogenous released from mitochondria or nucleus, constitutes a fundamental mechanism of host defense. The DNA-sensing enzyme cGAS binds to the cytosolic DNA in response and produces cGAMP, which in turn binds to and activates STING located on the endoplasmic reticulum (ER) membrane^53^. The STING complex subsequently activates downstream transcription factors through TBK1^37^. We recently reported that hDT806 can cause DNA damage and induce cell apoptosis in HNSCC^29^. In line with the DNA sensing function of the cytosolic STING complex, we found that hDT806 treatment significantly stimulated STING expression and phosphorylation of TBK1 in the HNSCC cells. Activation of STING via binding of its ligand results in stimulating type 1 IFN production and cytokine secretion, such as CXCL10. The secreted IFN-I may function in a paracrine or autocrine manner to engage its receptor IFNAR1^53^. As a cell-intrinsic defense mechanism highly conserved in almost all cell types, IFN-I upon binding to IFNAR1 triggers MX1 expression, a sensitive surrogate marker for IFNAR1 signaling activation. Indeed, we found that hDT806 treatment induced a significant increase in CXCL10 and MX1 levels in HNSCC cells, as determined by western blot analysis. Moreover, IHC analysis revealed an increase in IFNA1, IFNB, CXCL10, and MX1 in HNSCC xenograft tumors. STING signaling has been shown to activate NF-κB and MAP kinases, including p38 and ERK1/2 phosphorylation in mouse bone marrow macrophages and other cell types^53^. Immunotoxin was reported to activate p38 as well as NF-κB p65 in mesothelin-expressing tumor cells^39^. We recently demonstrated that hDT806 increases ERK1/2 phosphorylation in HNSCC^29^. Here, we also found hDT806 can activate p38 and p65 by phosphorylation. We further confirmed the involvement of STING-IFN-I axis by genetic modification of the HNSCC cells. Overexpression of STING in the HNSCC cells had effects on the STING-IFN-I axis effectors that resembled those seen with hDT806 treatment. Previous research in neuroblastoma^54^ as well as in lung cancer cells^55^ showed that potent STING activation promotes apoptotic cell death. Consistently, in HNSCC cells and xenograft tumors, we also found STING overexpression rendered sensitivity to cell apoptosis.

SOX2^48^ and MYC^56^ have both been found to be associated with HNSCC. The key transcription factor, SOX2, is known to promote the growth and invasion of HNSCC cells. SOX2 overexpression has been found as a common feature of HNSCC. For example, a meta-analysis showed that SOX2 is aberrantly expressed in head and neck cancer, and high SOX2 expression, in addition to high tumor grades, advanced TNM stages, lymph node metastasis and distant metastasis, predicts an unfavorable patient overall survival (OS)^45^. It was also shown that SOX2 may have important roles in the stemness and progression of HNSCC^47^. Importantly, SOX2 has been reported to dampen the immunogenicity of HNSCC by targeting the STING pathway for degradation^48^. Since hDT806 could downregulate SOX2, this may explain, at least partly, why the innate immune sensor STING pathway was upregulated by hDT806 treatment. However, the exact mechanism remains to be further examined. MYC overexpression is also frequently observed in HNSCC. MYC can promote immune-suppression via inhibition of tumor cell-intrinsic STING-IFN-I signaling in breast cancer^50^. In HNSCC, MYC inhibition recently was found to increased CD8^+^ T cell-recruiting chemokines by inducing the DNA damage related STING pathway to promote CD8^+^ T cell infiltration in mouse models^56^. In our present study, we did not observe any apparent changes in ALDH1/2 protein levels, a known CSC marker associated with HNSCC^51^. However, the downregulation of either SOX2 or MYC in HNSCC cells was statistically significant with hDT806 treatment compared to the vehicle treatment. The decrease of SOX2 or MYC in HNSCC by hDT806 was parallel to hDT806's stimulation of the tumor-intrinsic STING pathway, indicating there could be a crosstalk between these transcription factors and STING signaling that warrants further study. It’s intriguing that hDT806 treatment increased NF-kB activation through phosphorylation of the p65 subunit while decreasing the level of MYC in JHU-029 cells. In our recent publication^29^, we reported that hDT806 treatment leads to the inhibition of gene transcription. This is supported by our findings that hDT806 treatment strongly inhibits the phosphorylation of Ser2/5 as well as Ser7 of the RNA polymerase II subunit. In a previous study on hepatocellular carcinoma, we reported that transcription inhibition via a CDK7 inhibitor targeting RNA polymerase II drastically decreased MYC cellular level. It’s possible that hDT806-induced gene transcription inhibition may be related to the decreased MYC level. Immunotoxin’s well-known protein synthesis inhibition may contribute to this phenomenon. Further studies are needed to elucidate the mechanism behind these results.

A myriad of studies have implicated that STING is a promising innate immune target in cancer immunotherapy. Recently, a critical role of STING has been proposed to tumor surveillance, immune-mediated antitumor response, and tumor clearance^36^. Since STING is known to be a central mediator of innate and adaptive immunity, STING agonists have become a hot area of scientific exploration for patients with solid tumors^8^. Indeed, activation of the STING pathway in HNSCC has shown to be important for the innate immune system to sense tumors and initiate an IFN-I-driven program and ultimately lead to tumor-specific CD8^+^ T cells mobilization^48^. Clinical trials demonstrated that immunotoxin can markedly shrink or even erase tumors and prolong survival in some patients with HNSCC and other solid tumors. Observations from patients and studies in mouse models indicate that such remarkable efficacy was attributed to the immunotoxin-induced anticancer immunity^21^. Our data demonstrated hDT806 in both in vitro and in vivo settings can exploit tumor-intrinsic STING/IFN-I signaling to induce apoptosis and inhibit tumor cell growth in HNSCC. While the results show that hDT806 can stimulate a tumor-intrinsic innate immune response, the current study contains some inherent limitations. Our current study was not specifically designed to address the questions regarding the interaction between hDT806-induced STING-IFN-I axis and innate and adaptive immune responses. Instead, we employed an immuno-deficient NSG mouse model bearing xenograft tumors to investigate the essential role of the tumor-intrinsic STING-IFN-I axis in HNSCC tumor growth. This approach not only provides a platform for studying the crucial role of the STING-IFN-I axis but also effectively mitigates potential confounding effects arising from adaptive immune responses induced by hDT806.

## Conclusions

In conclusion, our study highlights the importance of the tumor-intrinsic STING-IFN-I signaling axis in mediating the anticancer effects of hDT806 in HNSCC. Given the frequent suppression of STING-IFN-I signaling in HNSCC patients^10^, the reactivation of this pathway emerges as a promising strategy to enhance the efficacy of cancer treatments. In our future studies, we will explore the potential of hDT806-activated tumor-intrinsic STING-IFN-I signaling to enhance both innate and adaptive immune responses against HNSCC. Overall, our findings provide valuable insights into the advancement of innovative immunotherapeutic strategies for treating HNSCC.

### Supplementary Information

Supplementary Figures.

## Data Availability

All data generated or analyzed during this study are included in this published article.
