# Supplementary material for: Recombinant immunotoxin induces tumor intrinsic STING signaling against head and neck squamous cell carcinoma
Source: Sci Rep. 2023 Oct 28;13:18476. doi: 10.1038/s41598-023-45797-7 (PMC10613212; doi:10.1038/s41598-023-45797-7)

Recombinant Immunotoxin Induces Tumor-intrinsic STING Signaling against Head and Neck Squamous Cell Carcinoma

Guiqin Xie[1,2*], Liang Shan[2], Cuicui Yang[1,2], Yuanyi Liu[3], Xiaowu Pang[1], Shaolei Teng[4], Tzyy-Choou Wu[5], and Xinbin Gu[1,2*]

[1]Department of Oral Pathology, Howard University, 600 W Street NW, Washington, DC 20059, USA; cuicui.yang@Howard.edu (C.Y); xpang@Howard.edu (X.P)

[2]Cancer Center, Howard University, 2041 Georgia Avenue NW, Washington, DC 20059, USA; shanliang1964@gmail.com

[3]Angimmune LLC, Rockville, MD 20855, USA; yyliu6@gmail.com

[4] Department of Biology, Howard University, 415 College St. NW, Washington, DC 20059, USA; shaolei.teng@howard.edu

[5]Pathology, Oncology, Obstetrics & Gynecology, and Molecular Microbiology &Immunology, Johns Hopkins University School of Medicine, MD 21287, USA; wutc@jhmi.edu

*****Correspondence: guiqin.xie@Howard.edu (G.X); xgu@Howard.edu (X.G)

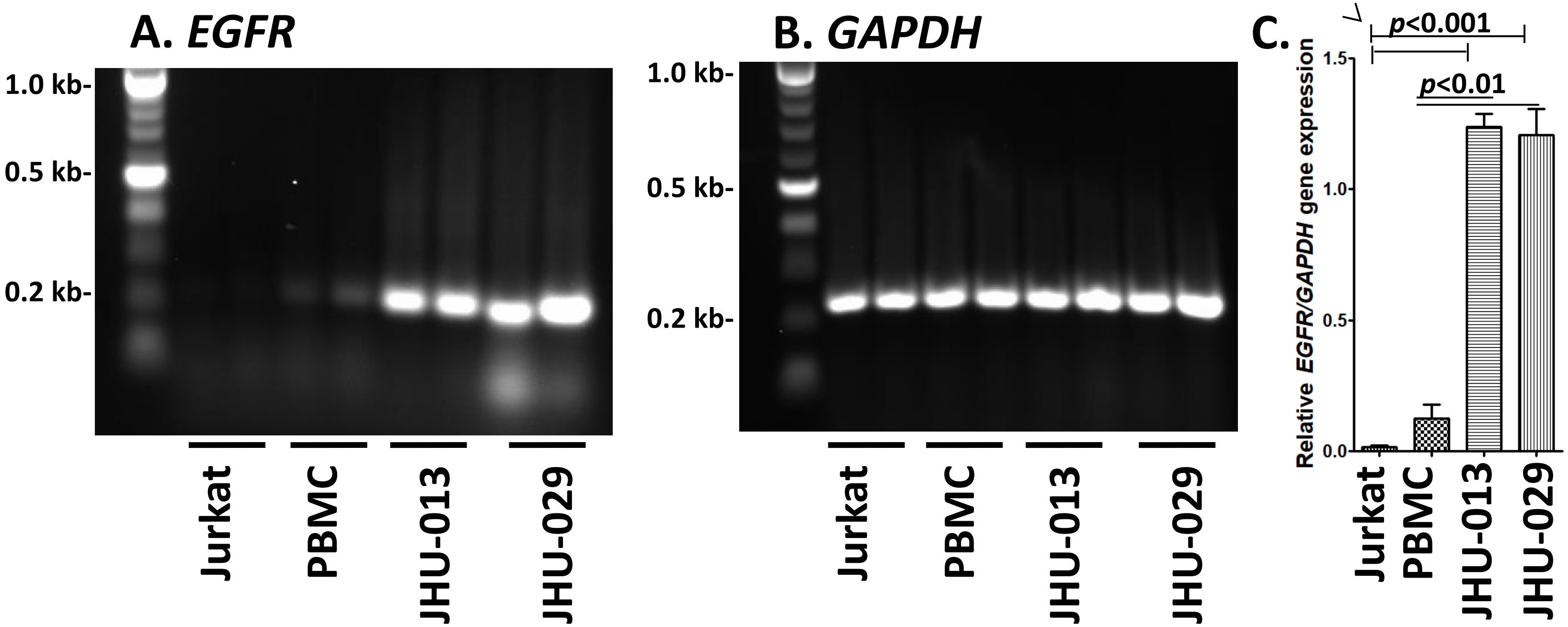

Supplementary Figure S1. *EGFR* and *GAPDH* gene expression. Gel electrophoresis of *EGFR* (A) and *GAPDH* (B) gene expression in Jurkat, human peripheral blood mononuclear cell (PBMC), JHU-013, and JHU-029 cells, presented in duplicate. (C) Relative *EGFR/GAPDH* gene expression in Jurkat, PBMC, JHU-013, and JHU-029 cells is shown as mean ± SEM (n=3), with *p* values indicated.

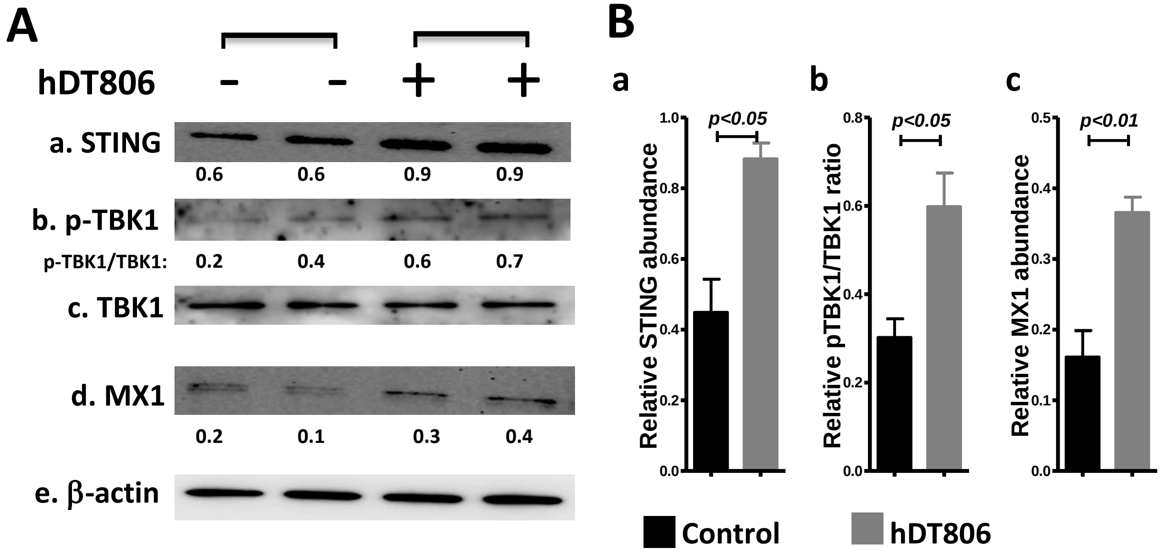

Supplementary Figure S2. hDT806 stimulates STING-IFN-I signaling in JHU-013 HNSCC cells. (A) Total protein extracts were prepared from the cells treated with vehicle or hDT806 (20 nM). Western blot analysis was performed for STING (a), p-TBK1 (b), TBK1 (c), MX1 (d), and β-actin (e) in the JHU-013 HNSCC cells treated with vehicle or hDT806 for 48 h. (B) Protein band intensities of STING (a) and MX1 (c) relative to the corresponding β-actin, and the ratio of p-TBK1 /TBK1 (b) were quantified for comparisons between the vehicle-treated cells and the hDT806-treated cells. Data from three or four independent experiments are presented as mean ± SEM, with *p* values indicated.

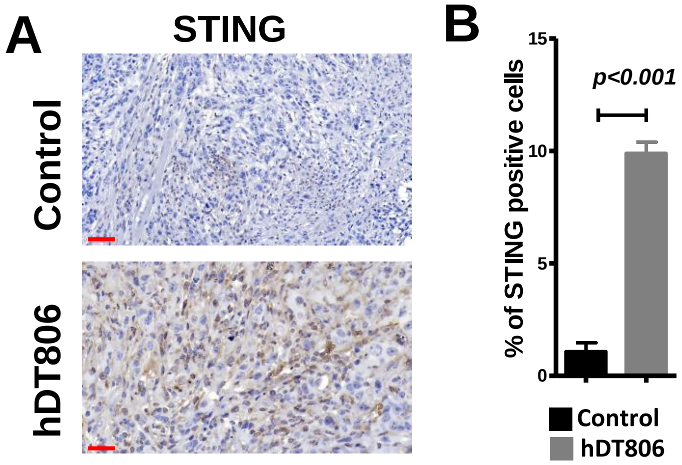

Supplementary Figure S3. hDT806 treatment increases STING expression in GL261vIII mouse glioblastoma models. GL261vIII mouse glioblastoma models shows a particular sensitivity to hDT806 in our previous studies (Meng et al., 2015)[28]. In line with the effects of hDT806 in HNSCC xenograft models, indeed, IHC analysis of the GL261vIII tumors revealed a dramatic increase in STING-positive cells in the hDT806-treated compared to the vehicle-treated tumors, from 1.1 ± 0.4% to 9.9 ± 0.5% (n=4, $p<0.001$). Inset scale bars: 30 µM.

Presentation of Original Immunoblotting and Gel Electrophoresis Data in Figures (Figures 1, 3, 4, 5, and 6; Supplementary Figures S1 and S2)

Figure 1Aa. JHU-029 (STING)

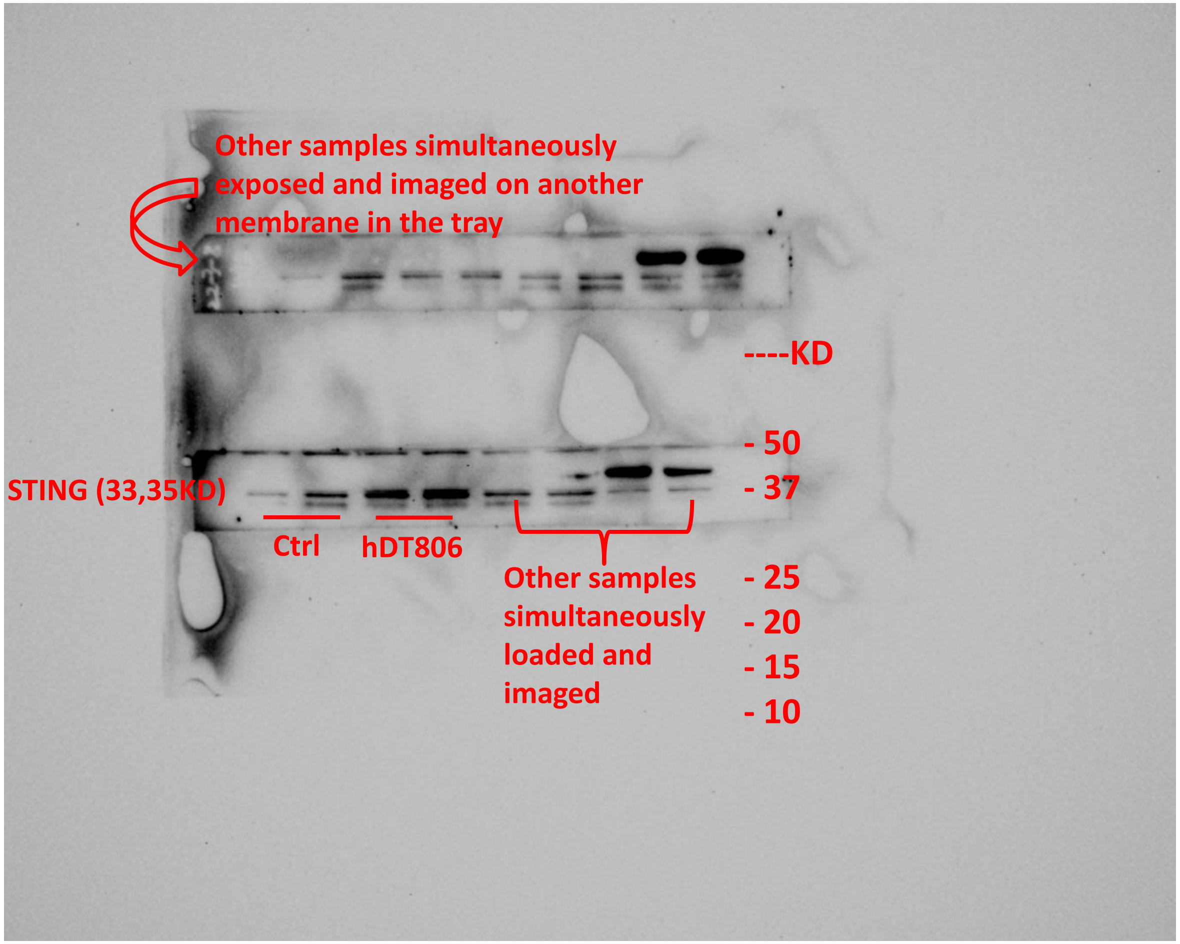

Figure 1Ab. JHU-029 (p-TBK1)

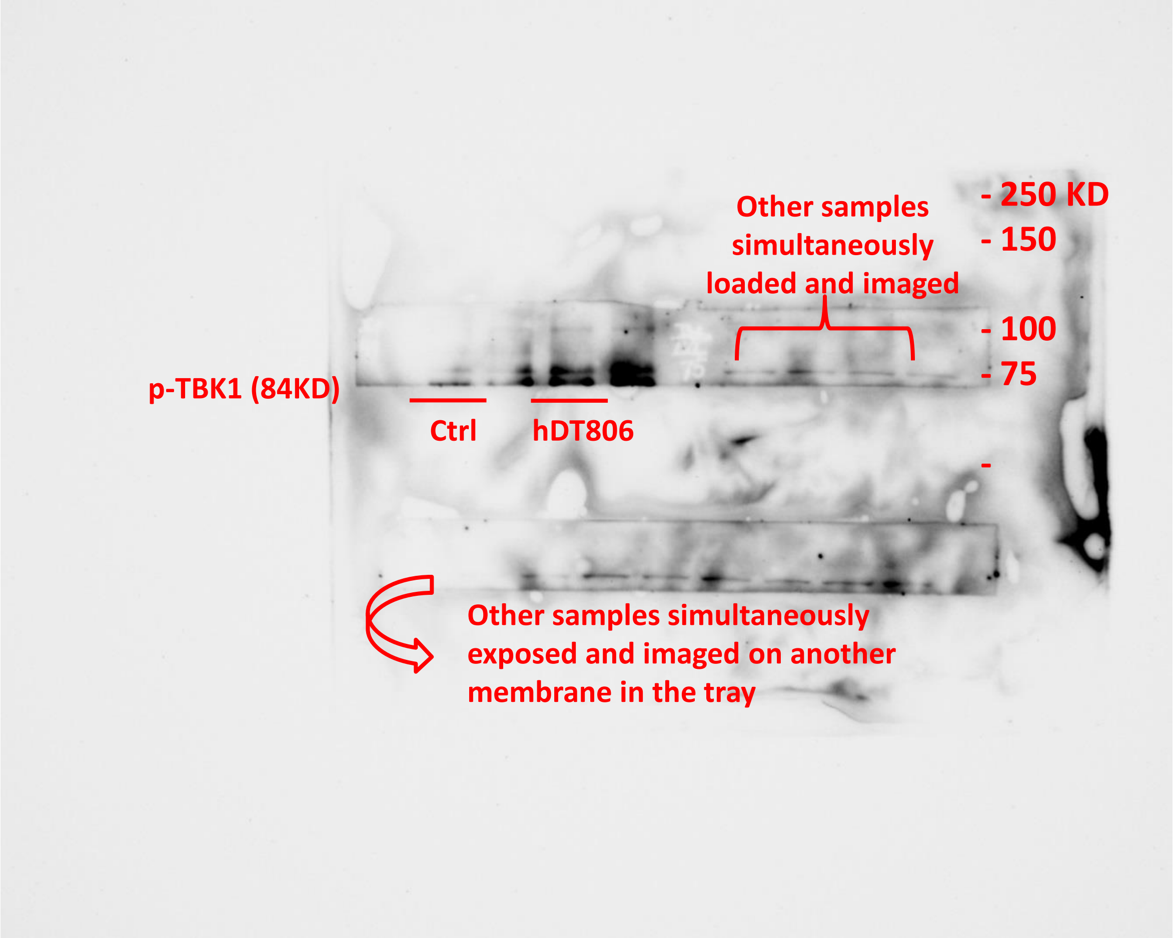

Figure 1Ac. JHU-029 (TBK1)

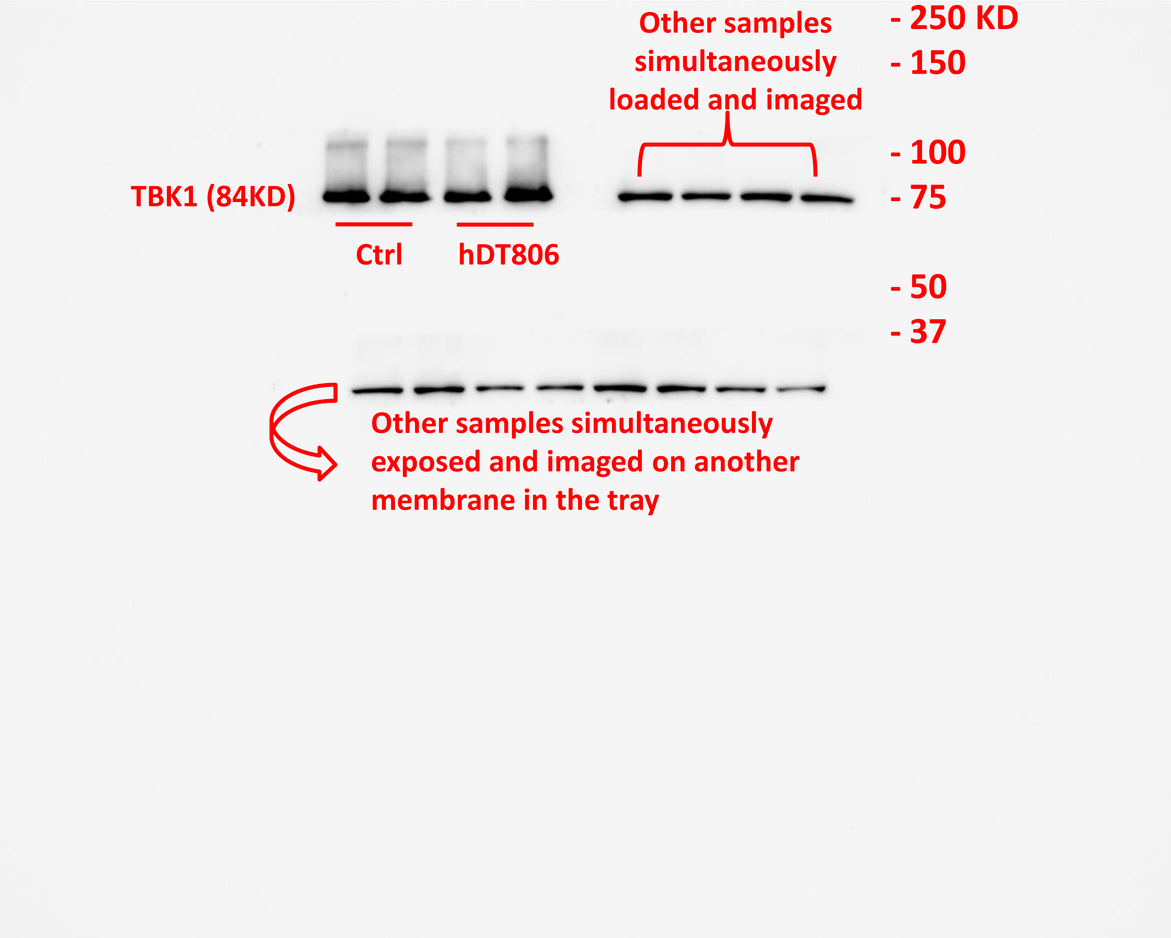

Figure 1Ad. JHU-029 (CXCL10)

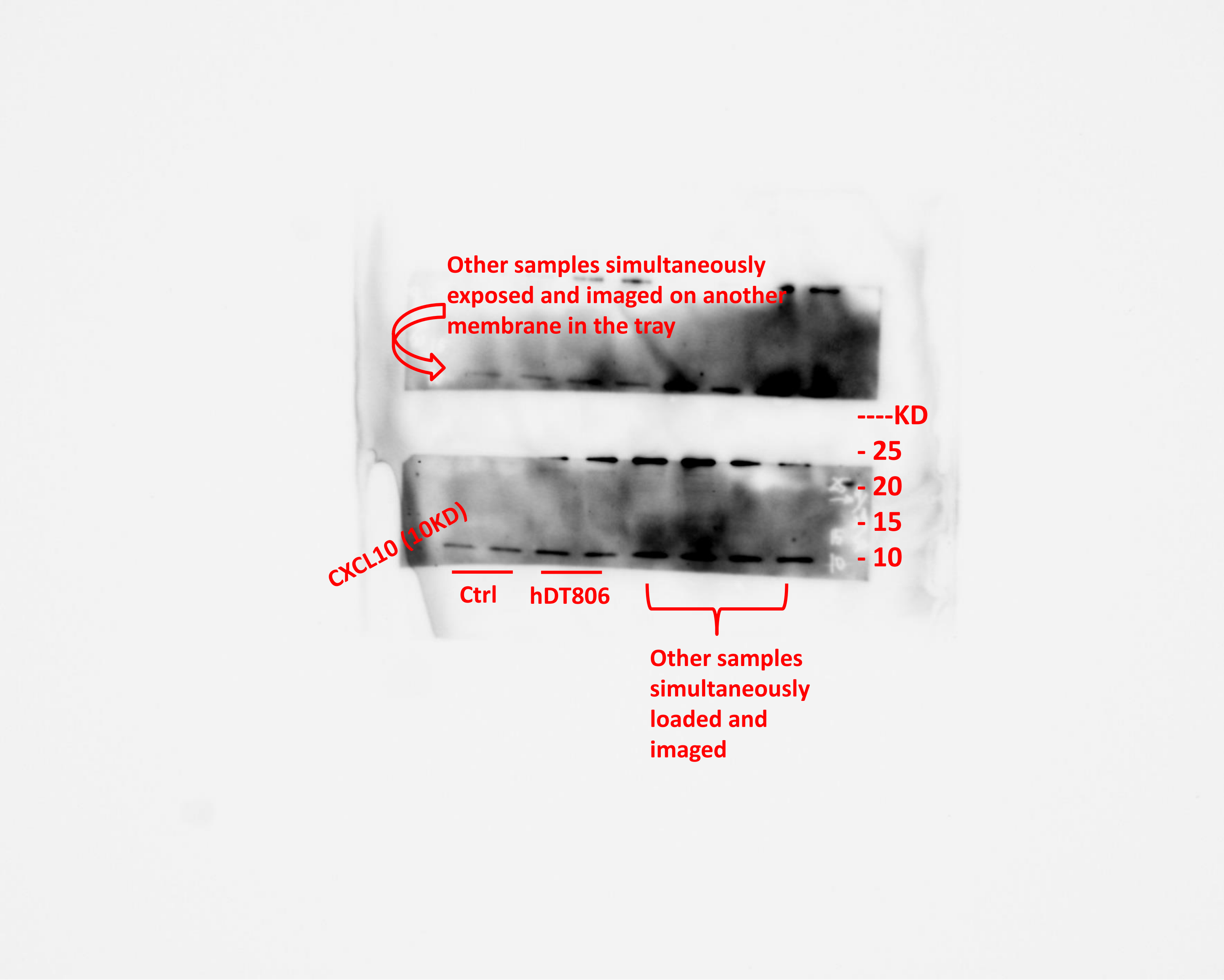

Figure 1Ae. JHU-029 (MX1)

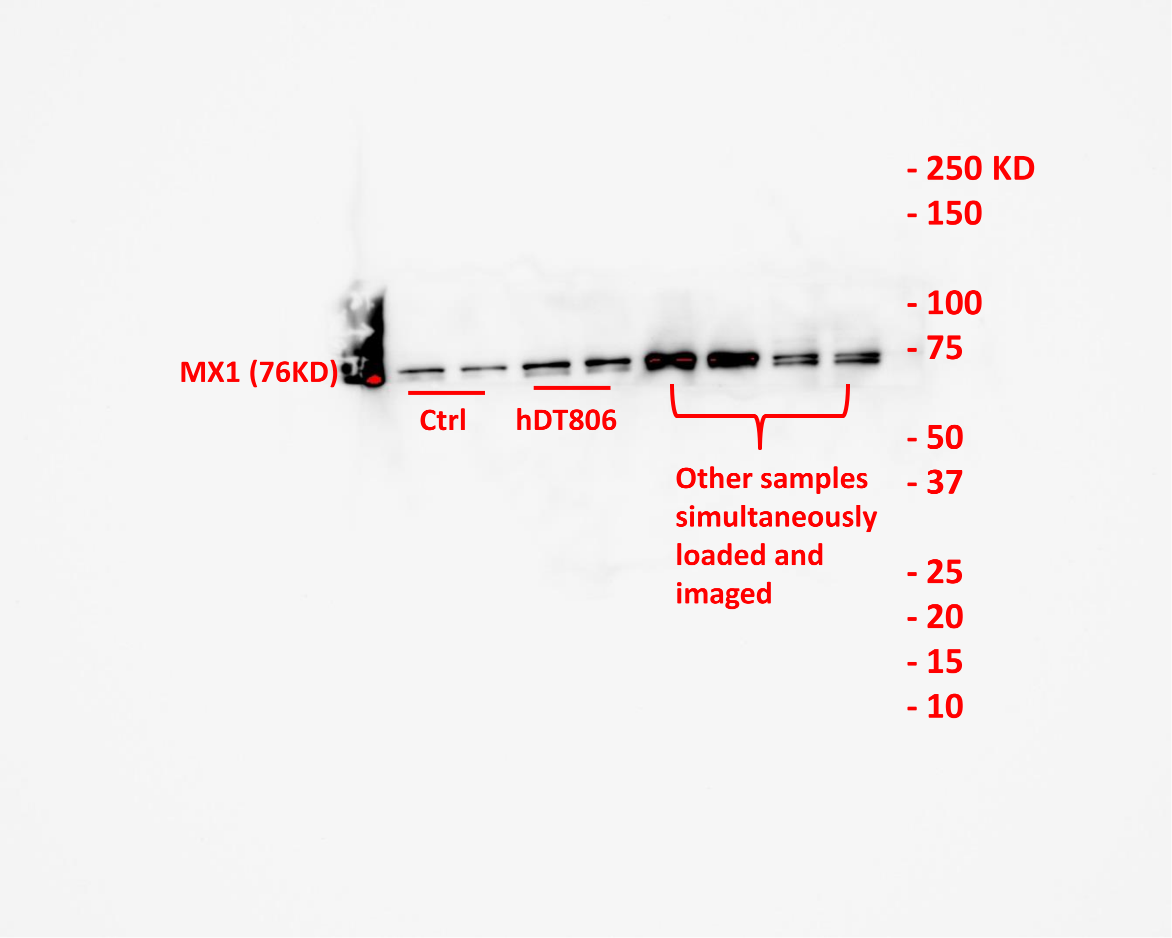

Figure 1Af. JHU-029 (Actin)

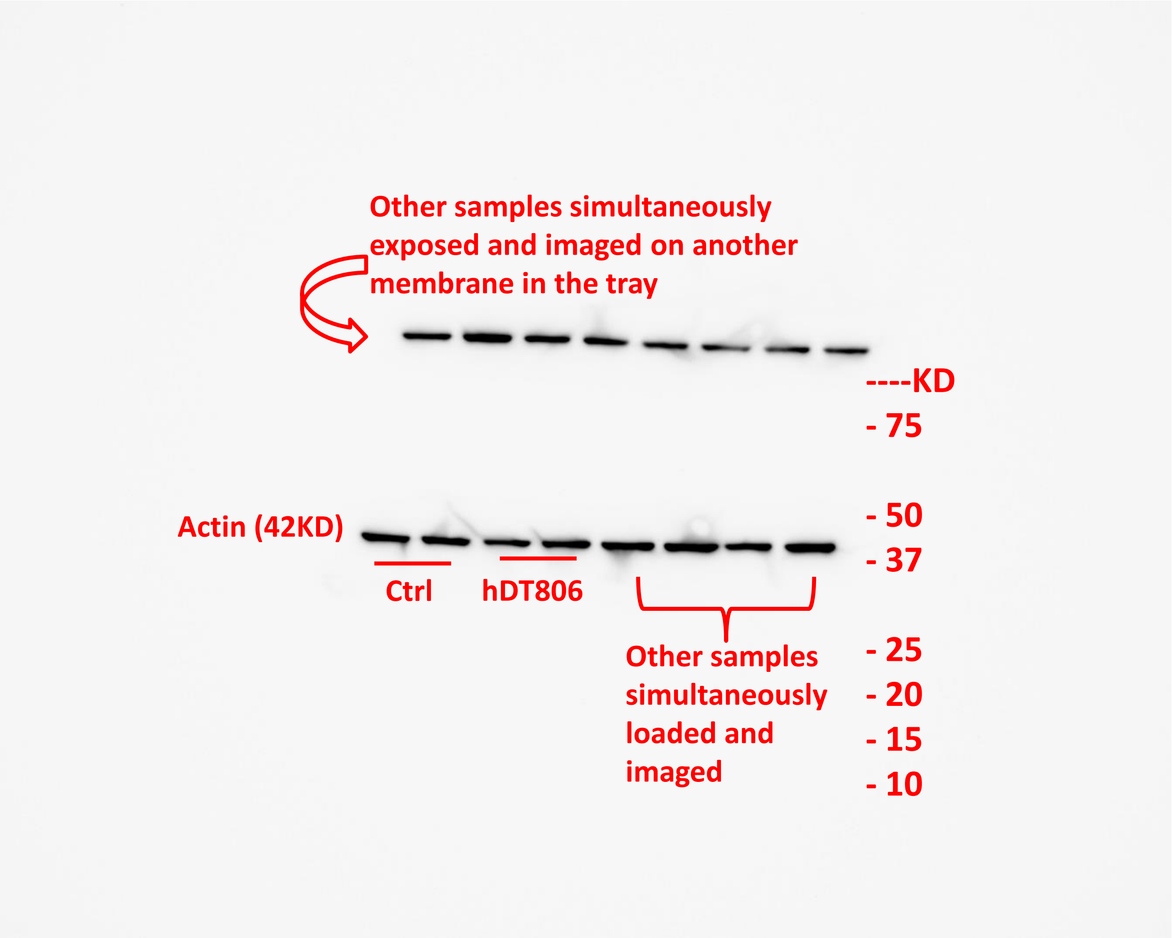

Figures 3Aa and 3Ac. JHU-029 (p-p38; p-p65)

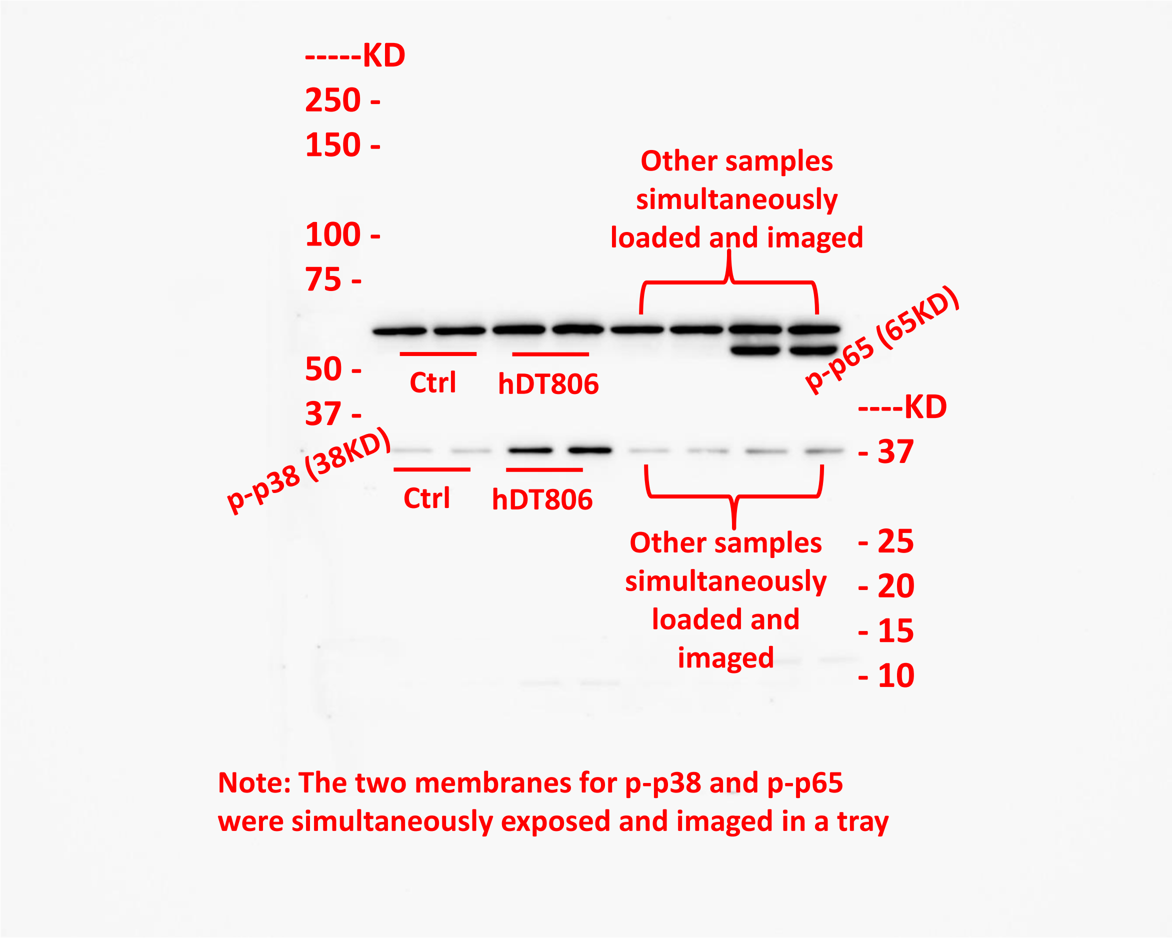

Figure 3Ab. JHU-029 (p38)

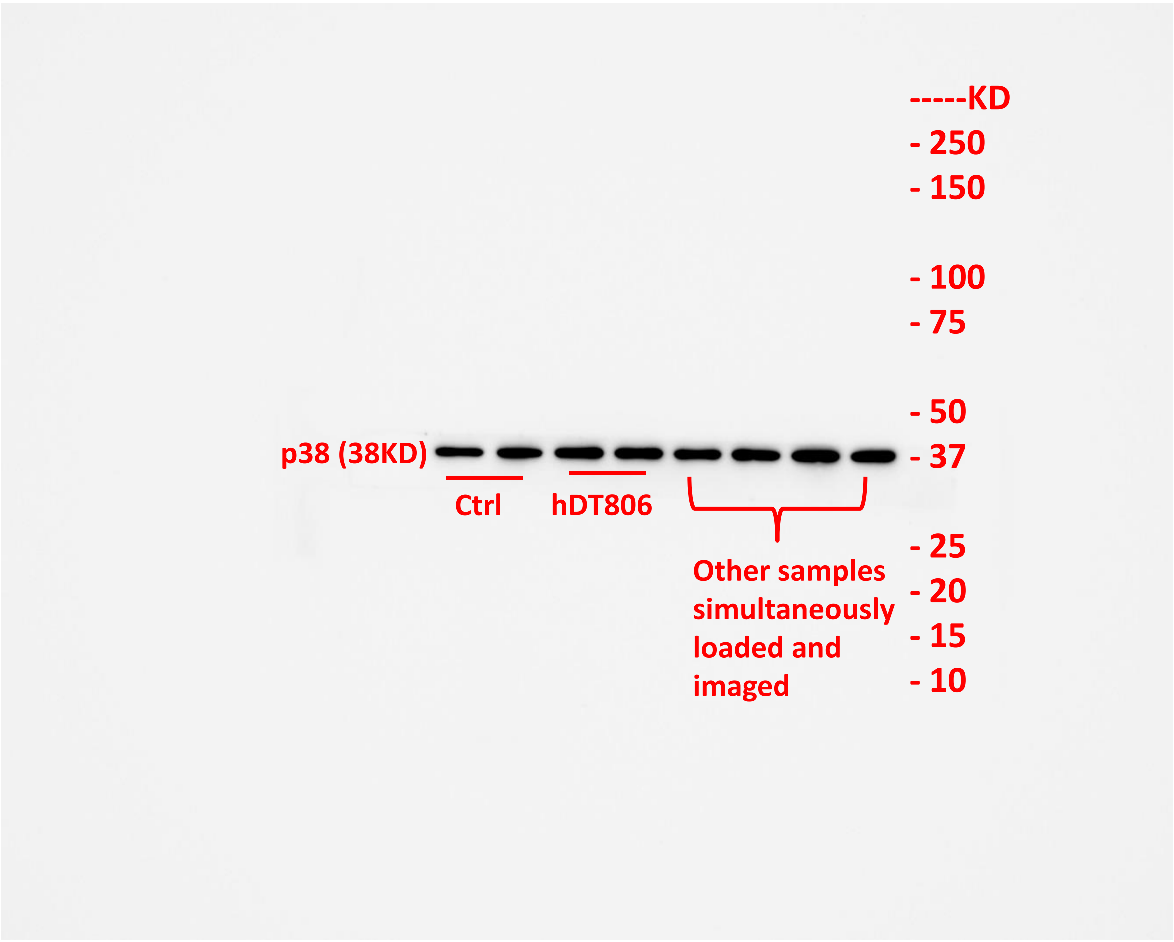

Figure 3Ad. JHU-029 (p65)

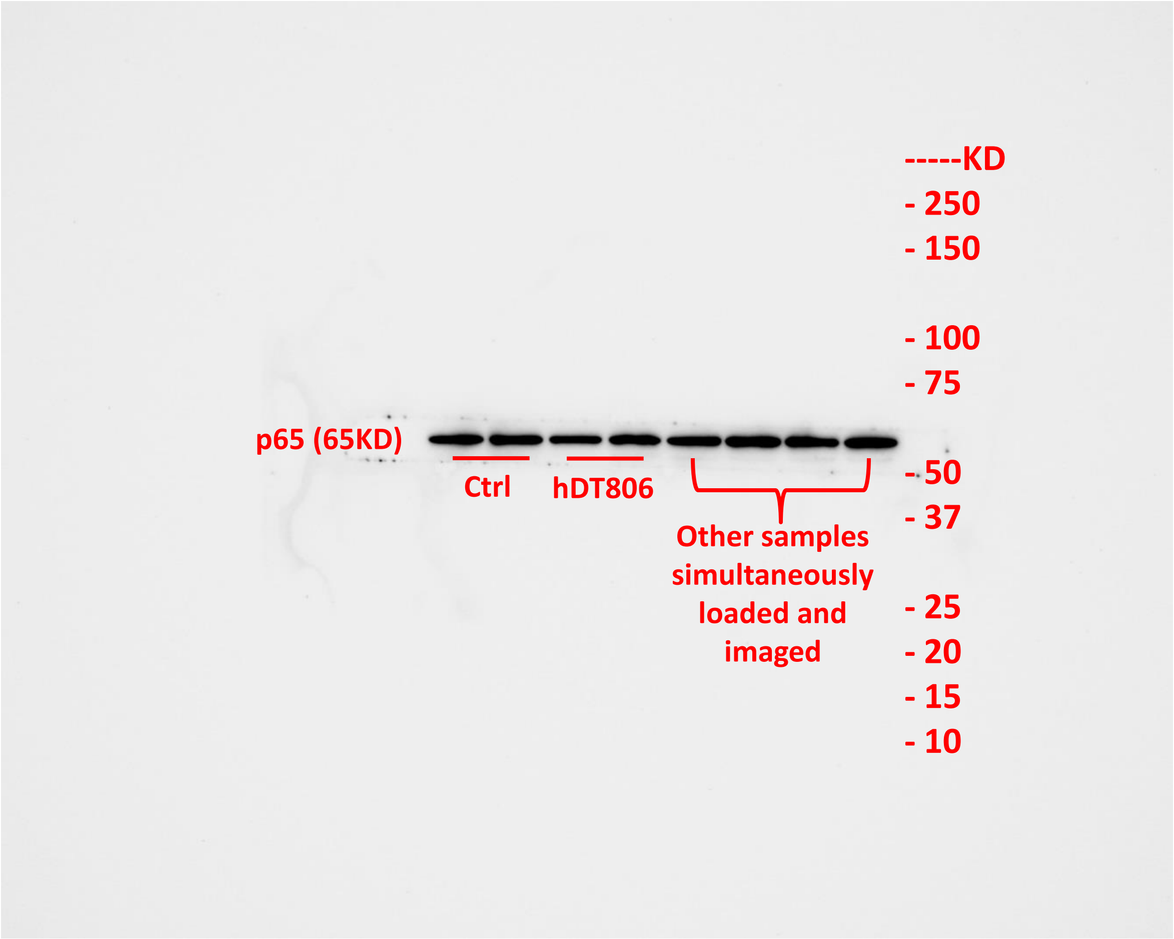

Figure 3Ae. JHU-029 (PD-L1)

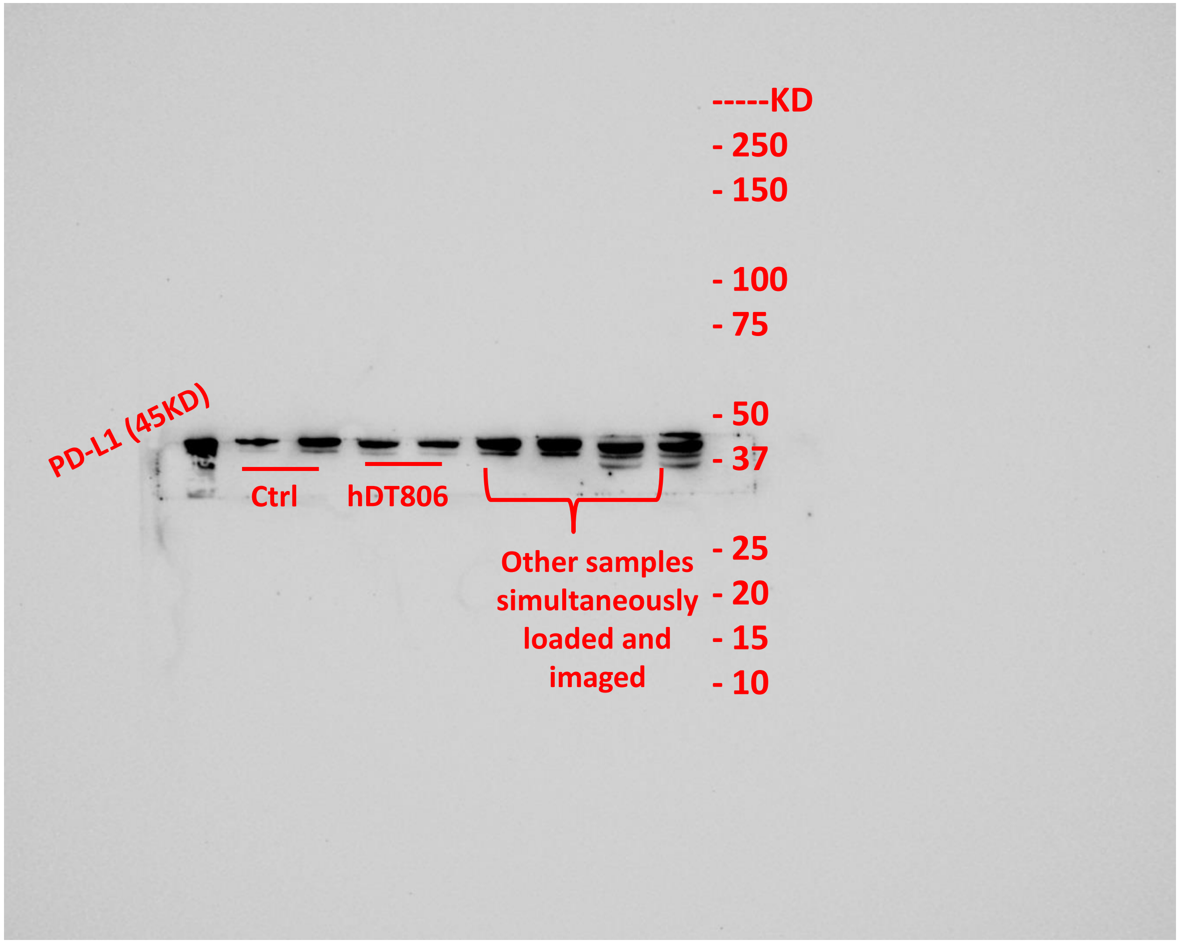

Figure 3Af. JHU-029 (Actin)

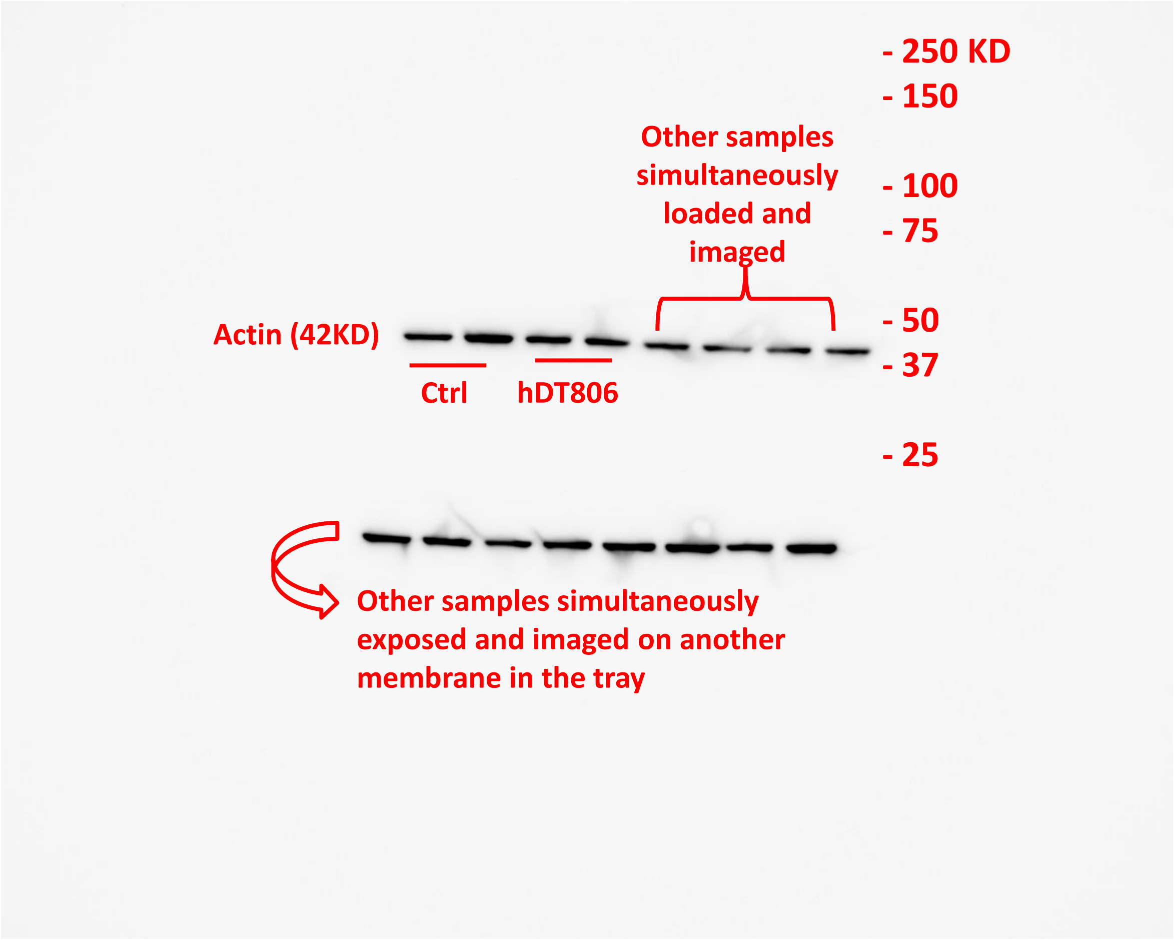

Figure 4Aa. JHU-029 (SOX2)

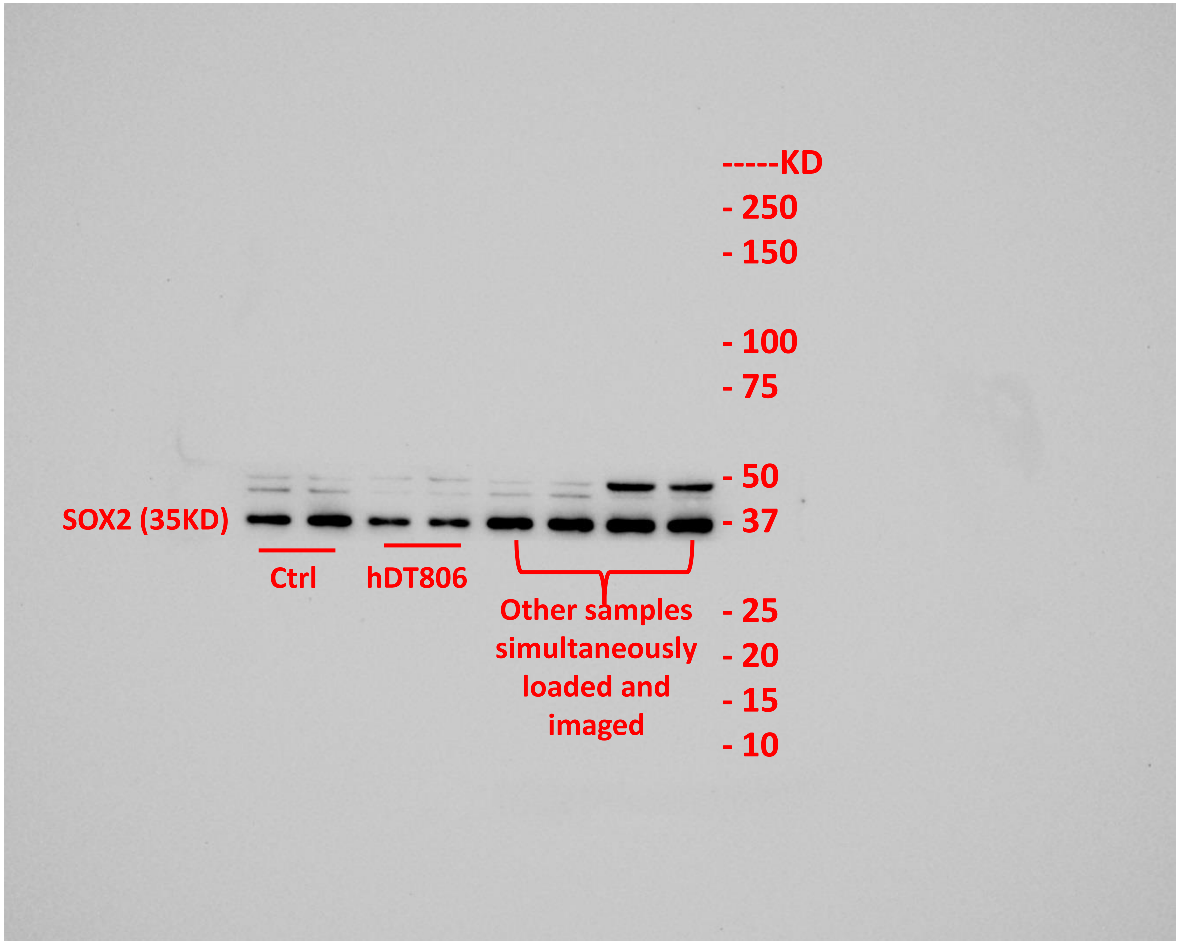

Figure 4Ab. JHU-029 (MYC)

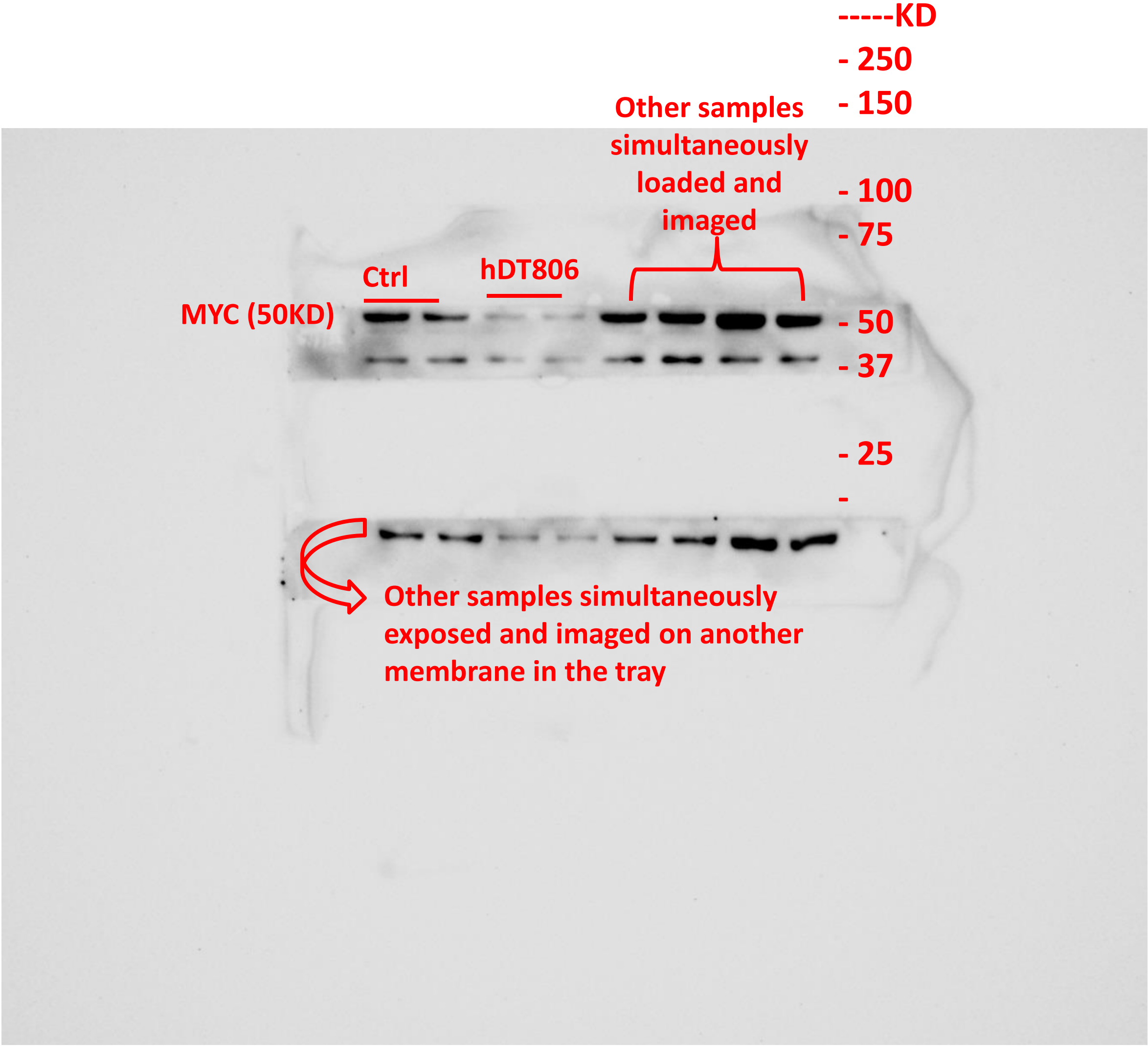

Figure 4Ac. JHU-029 (ALDH1/2)

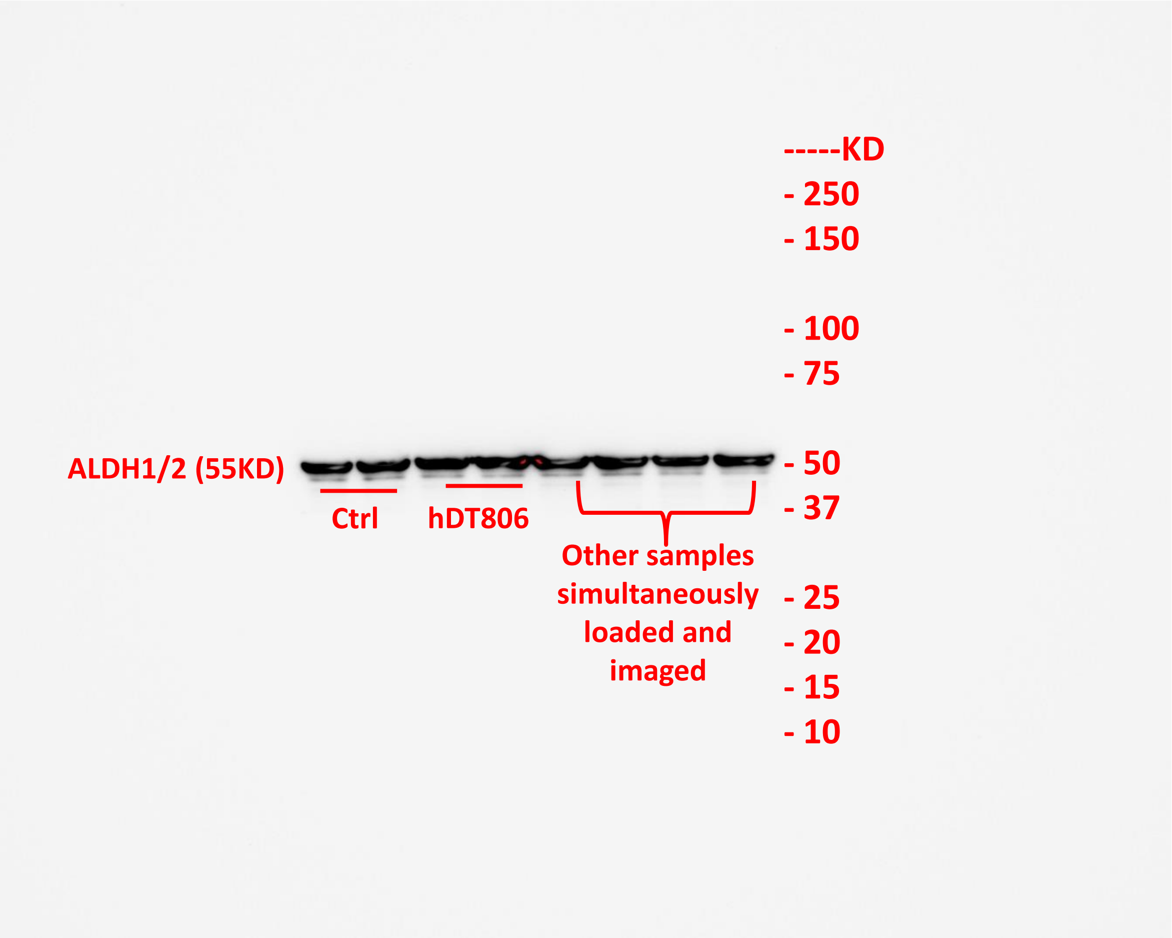

Figure 4Ad. JHU-029 (Actin)

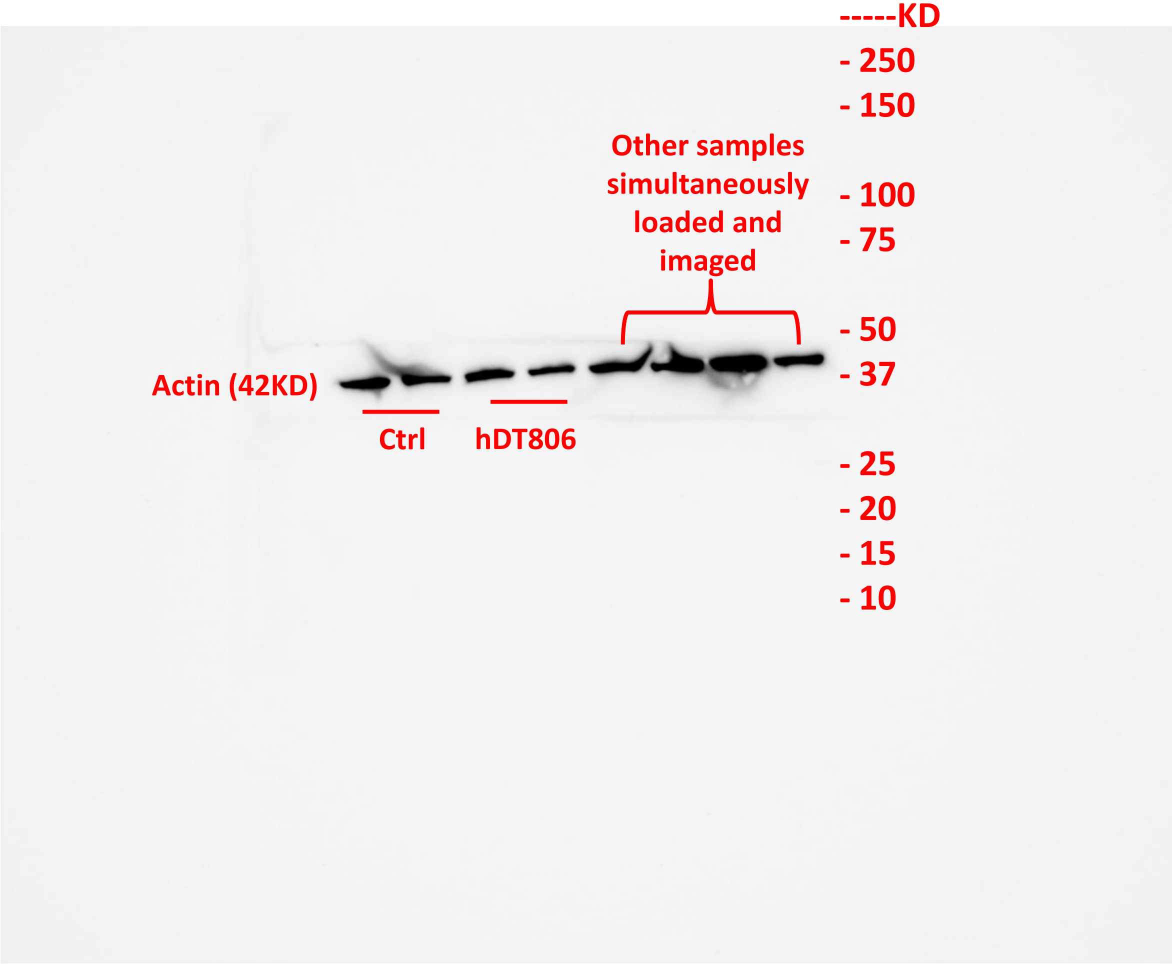

Figure 5Aa. JHU029-STING vs. JHU029-Ctrl (STING)

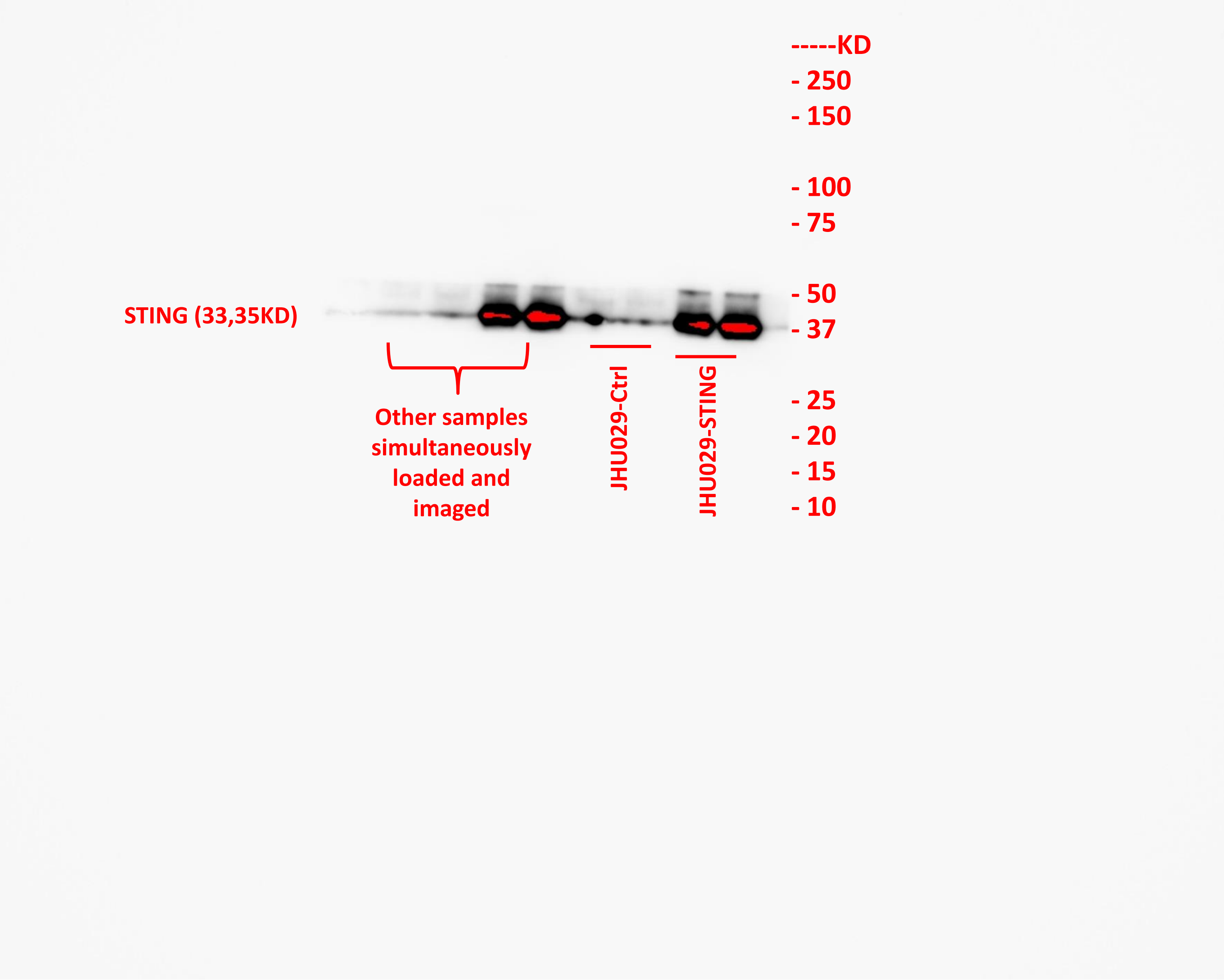

Figure 5Ab. JHU029-STING vs. JHU029-Ctrl (p-TBK1)

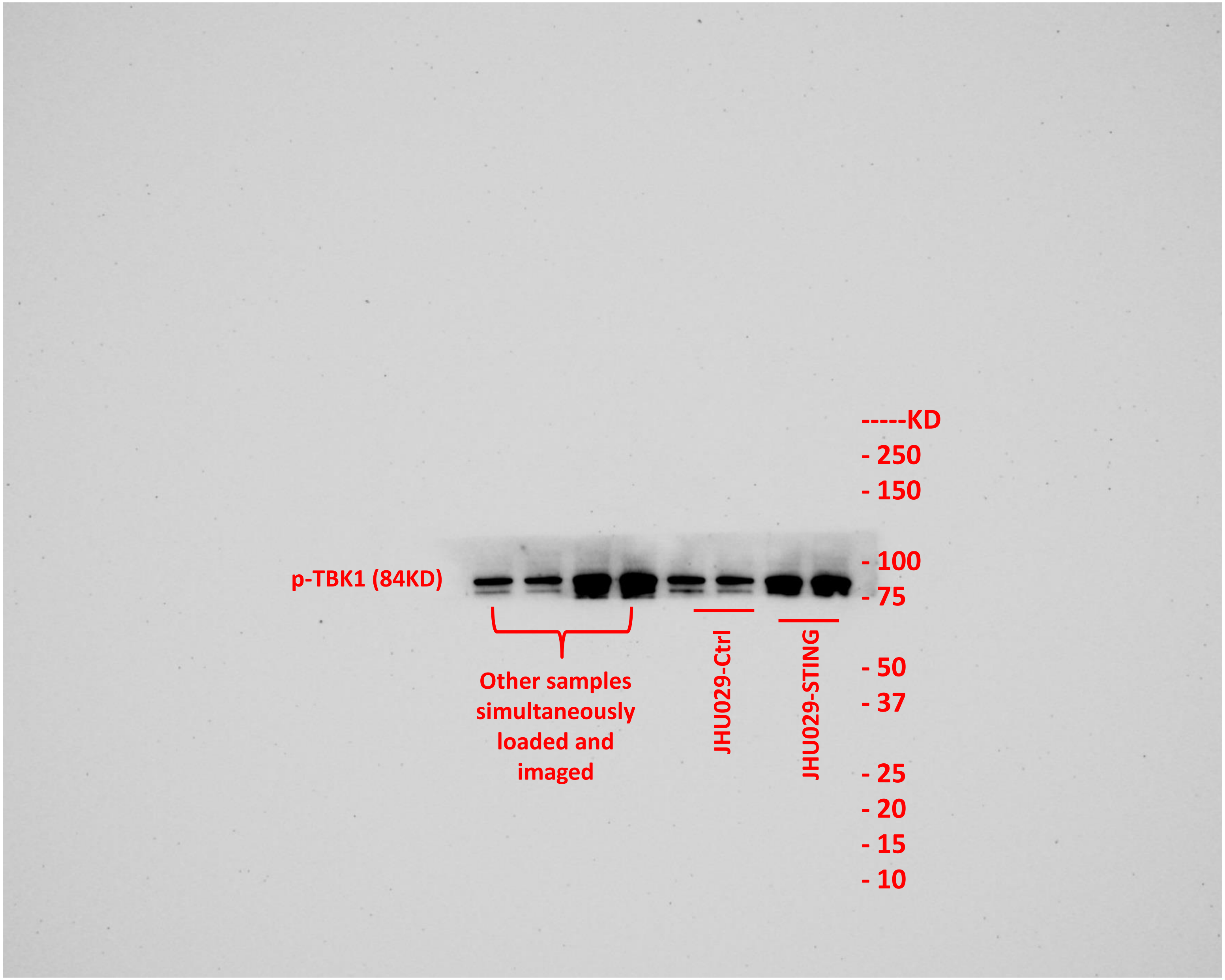

Figure 5Ac. JHU029-STING vs. JHU029-Ctrl (TBK1)

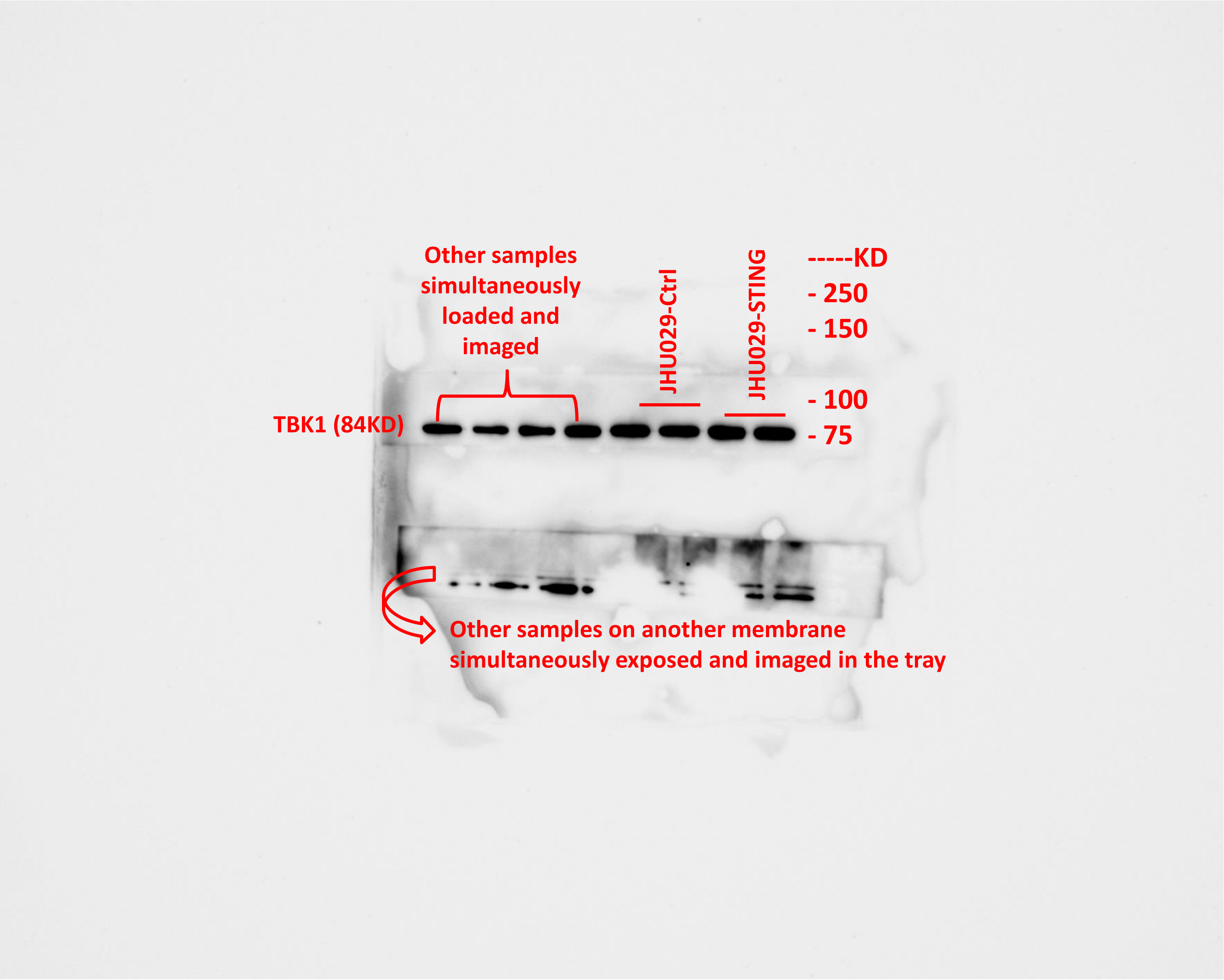

Figure 5Ad. JHU029-STING vs. JHU029-Ctrl (p-p38)

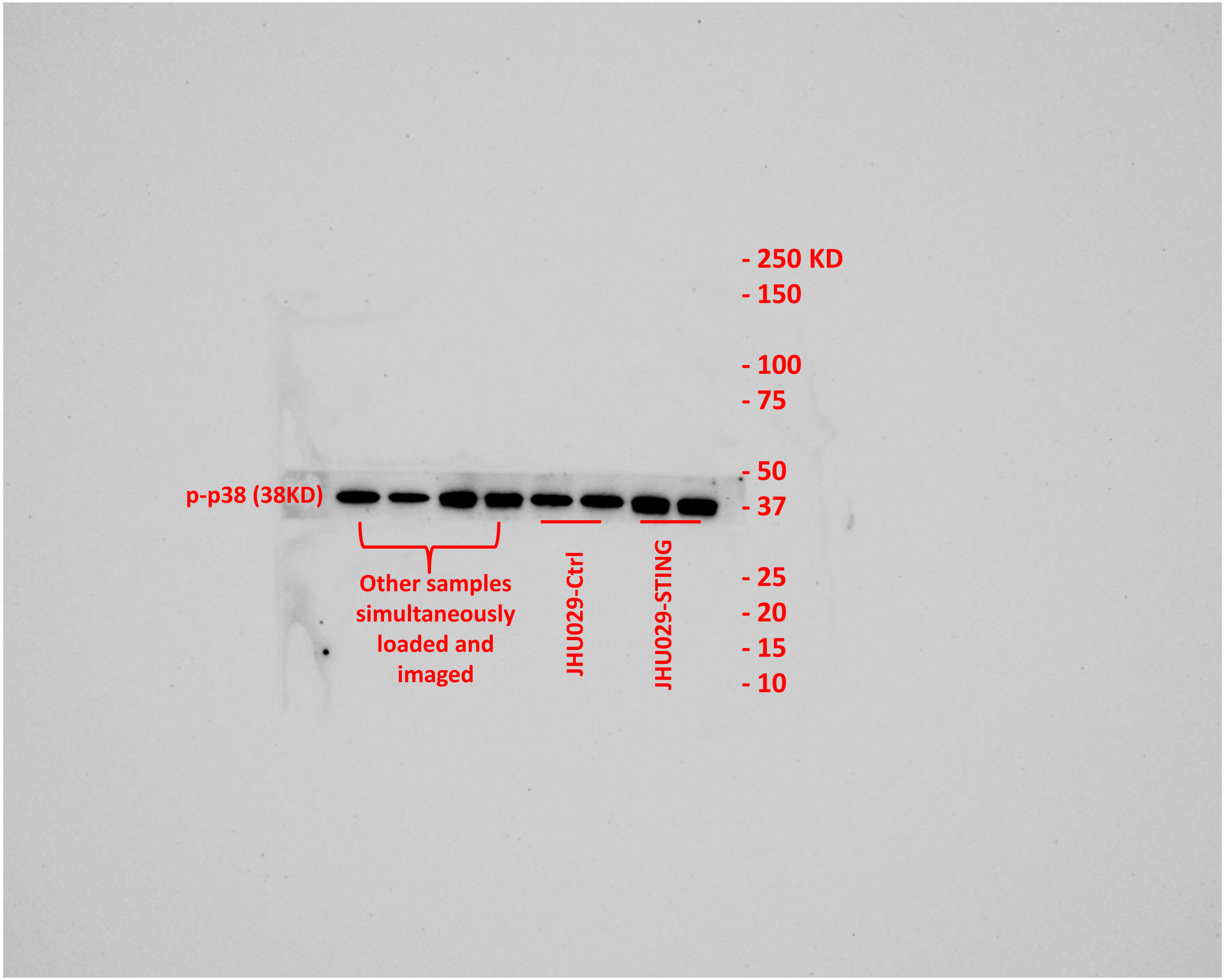

Figure 5Ae. JHU029-STING vs. JHU029-Ctrl (p38)

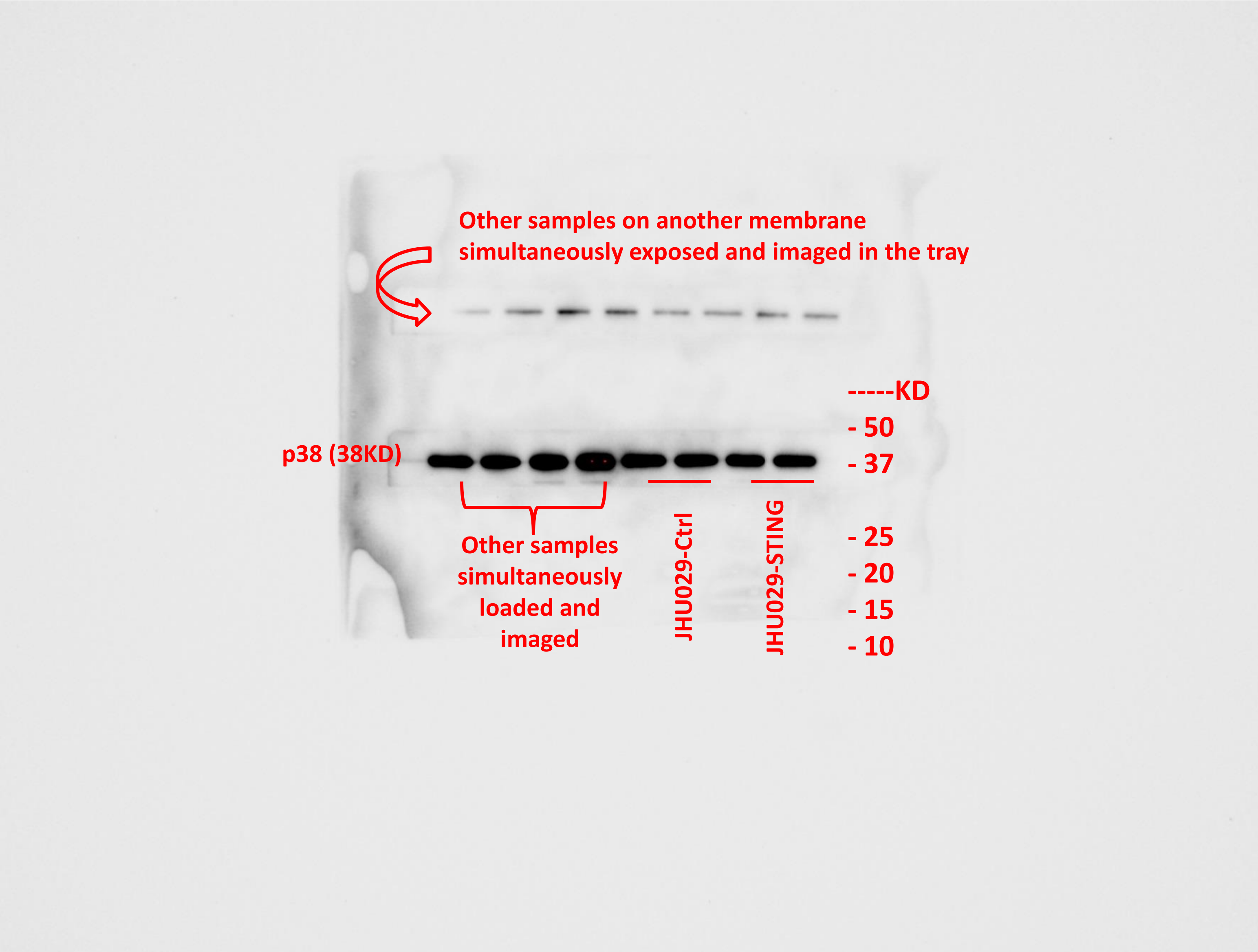

Figure 5Af. JHU029-STING vs. JHU029-Ctrl (IFNB)

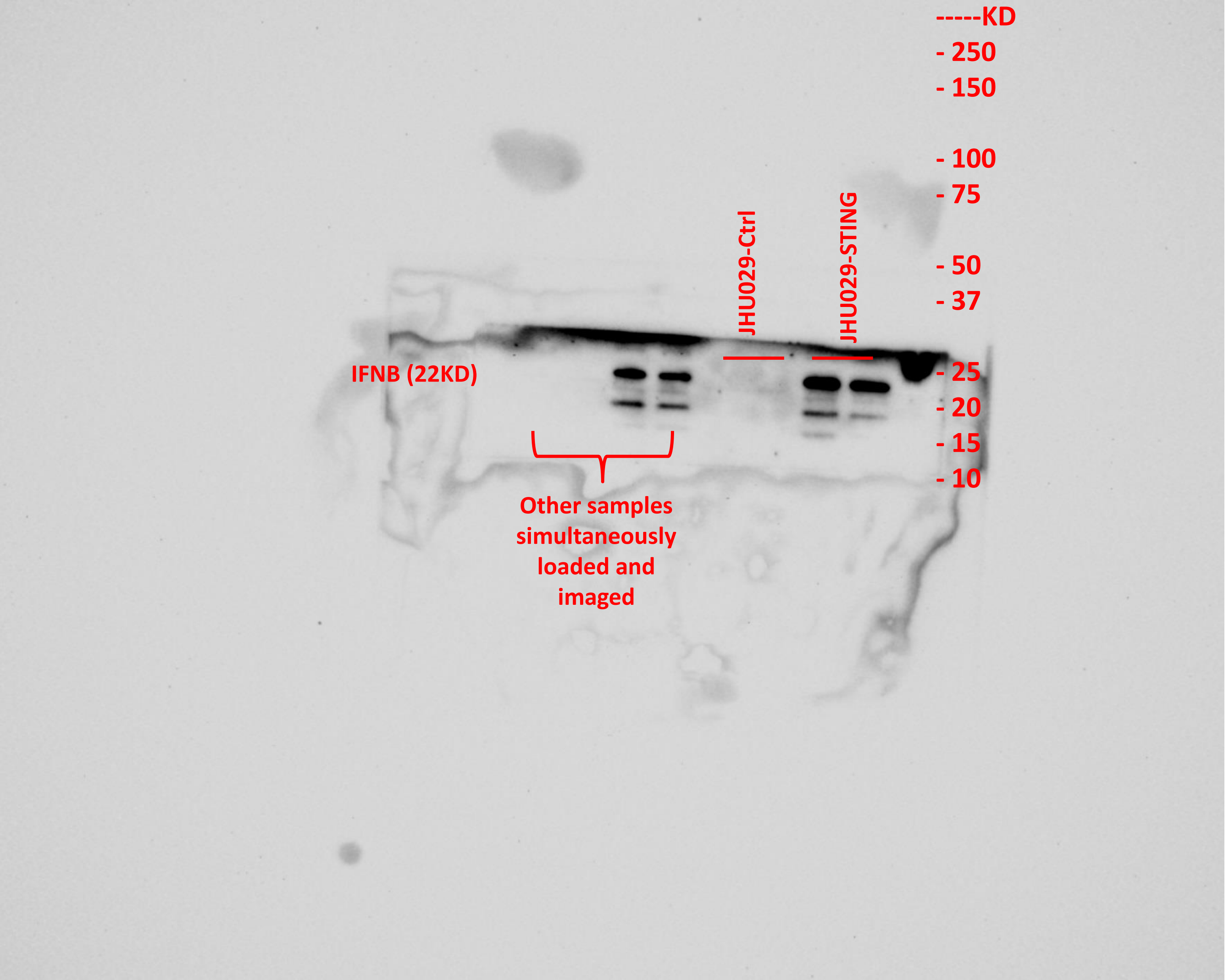

Figure 5Ag. JHU029-STING vs. JHU029-Ctrl (CXCL10)

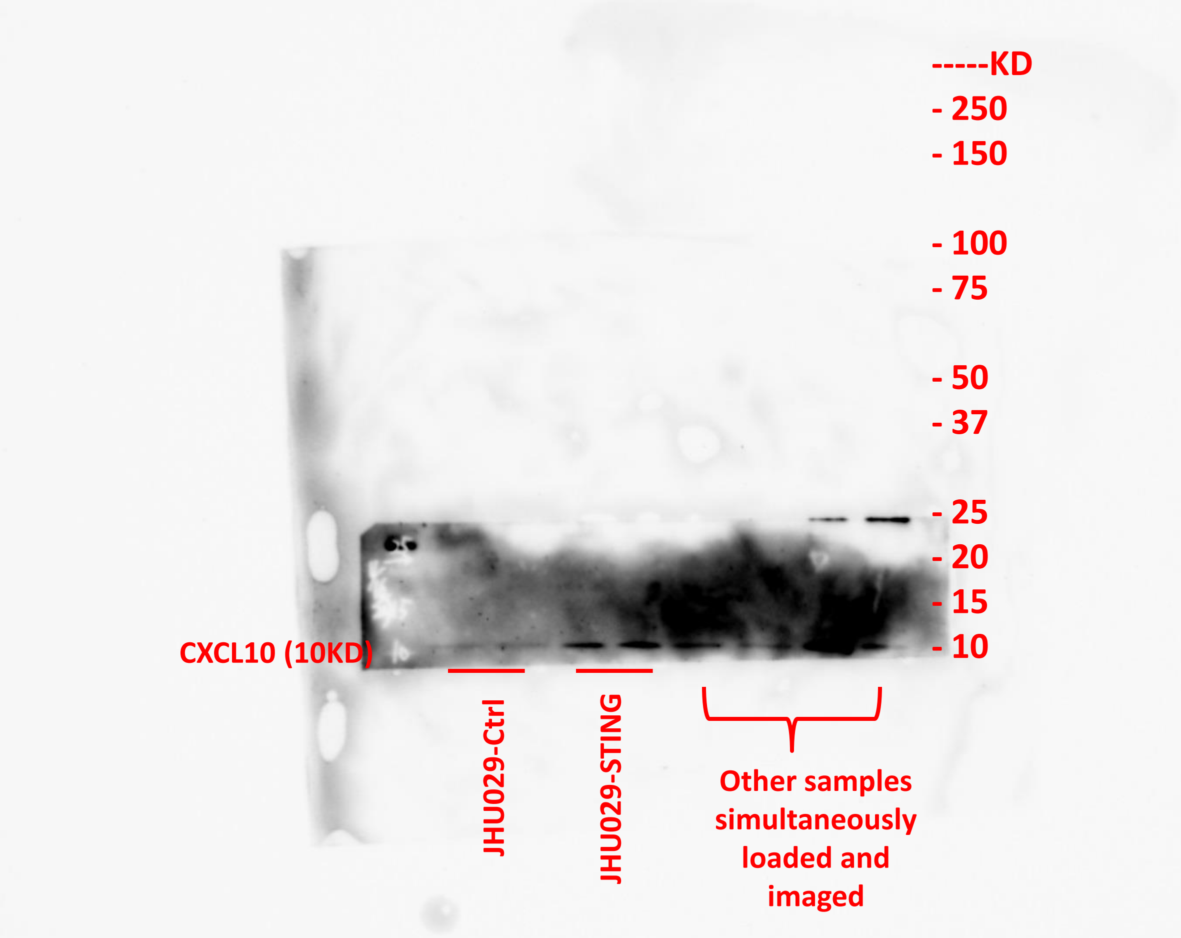

Figure 5Ah. JHU029-STING vs. JHU029-Ctrl (MX1)

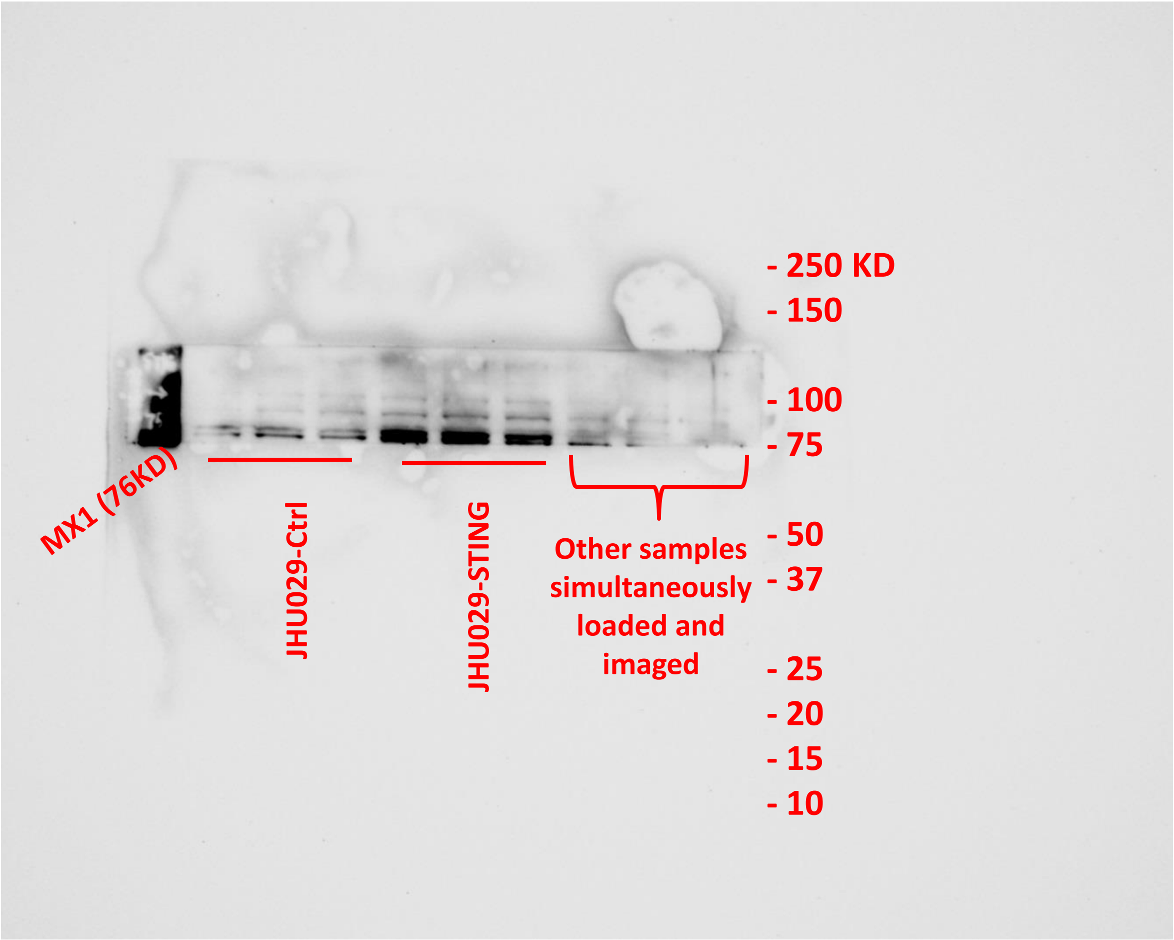

Figure 5Ai. JHU029-STING vs. JHU029-Ctrl (Actin)

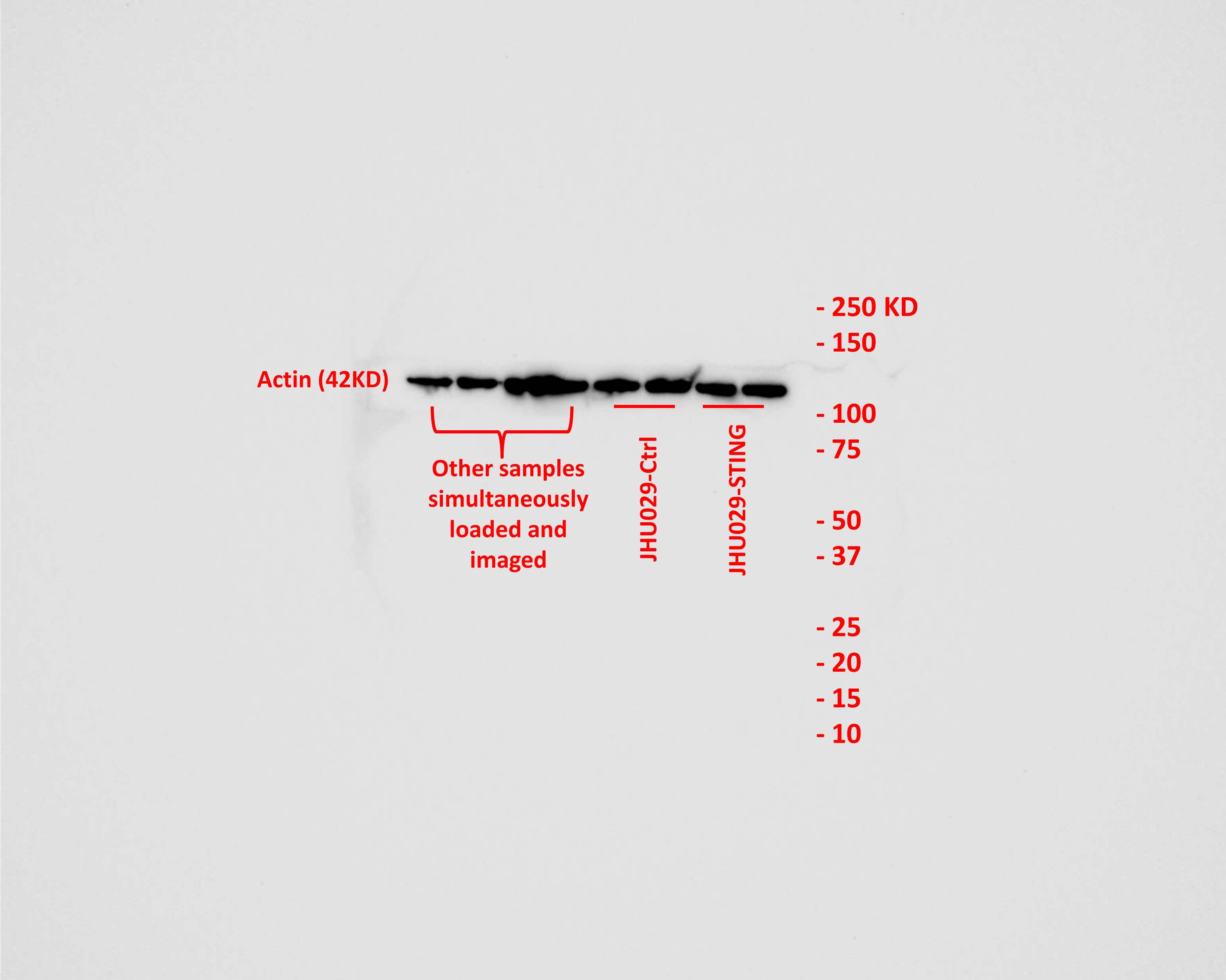

Figure 6Ca. JHU029-STING vs. JHU029-Ctrl (PARP/cleaved PARP)

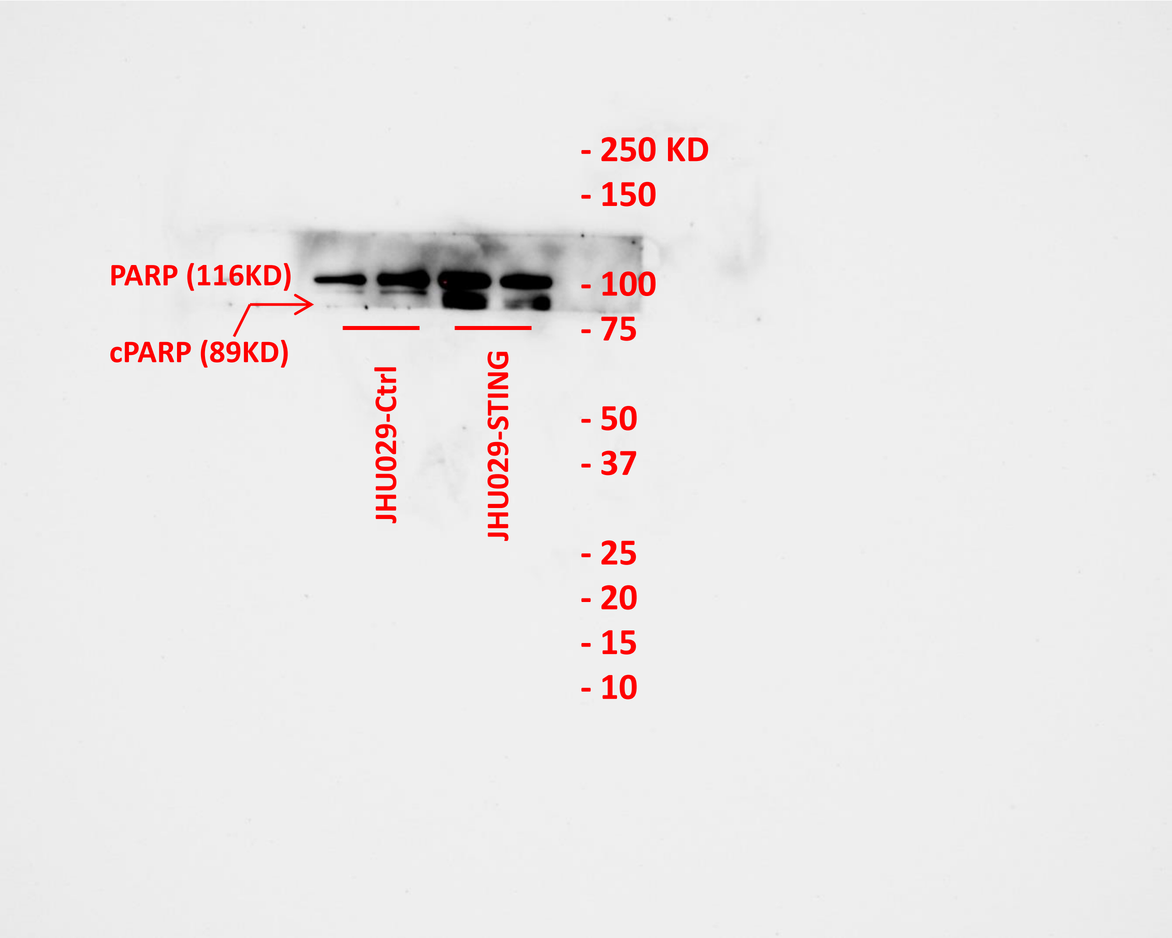

Figure 6Cb. JHU029-STING vs. JHU029-Ctrl (Caspase-9)

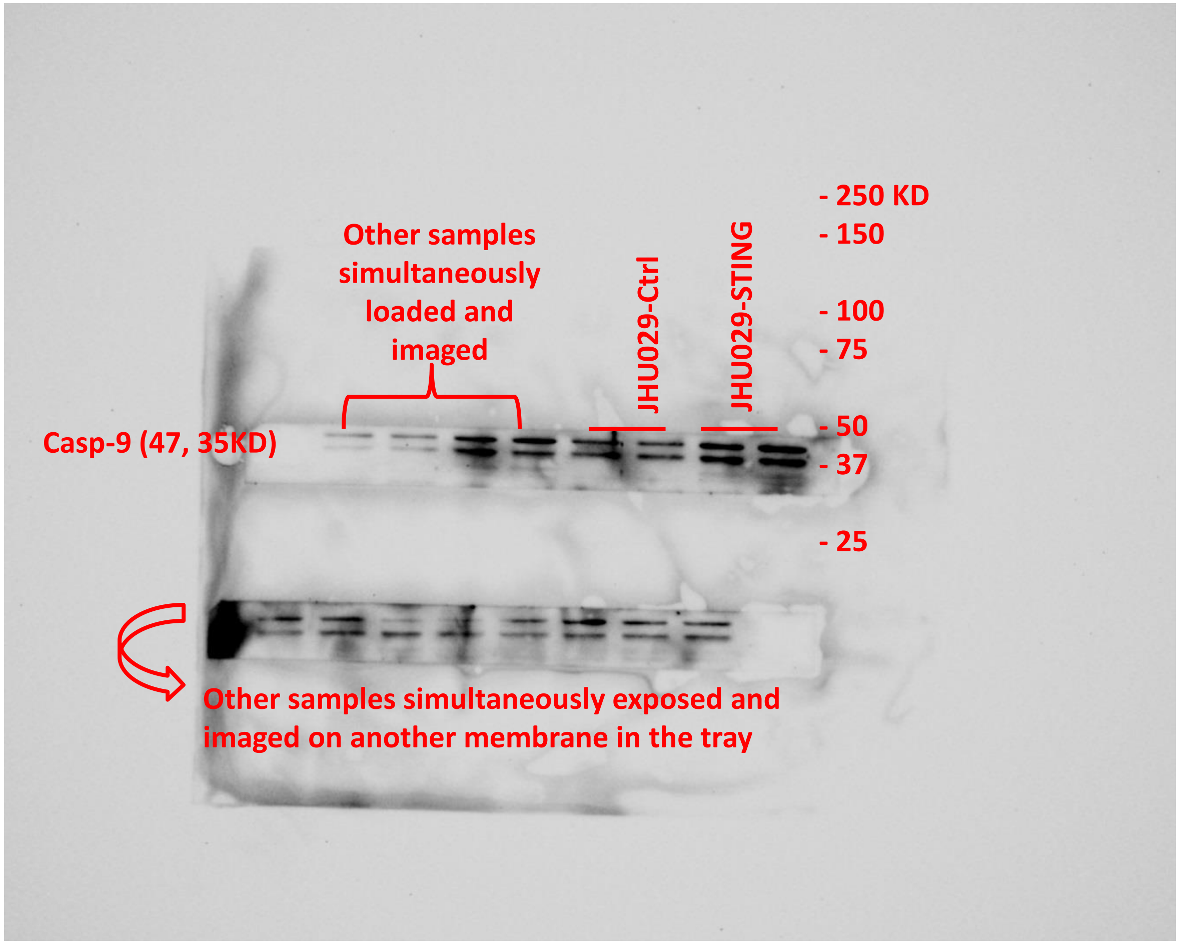

Figure 6Cc. JHU029-STING vs. JHU029-Ctrl (Actin)

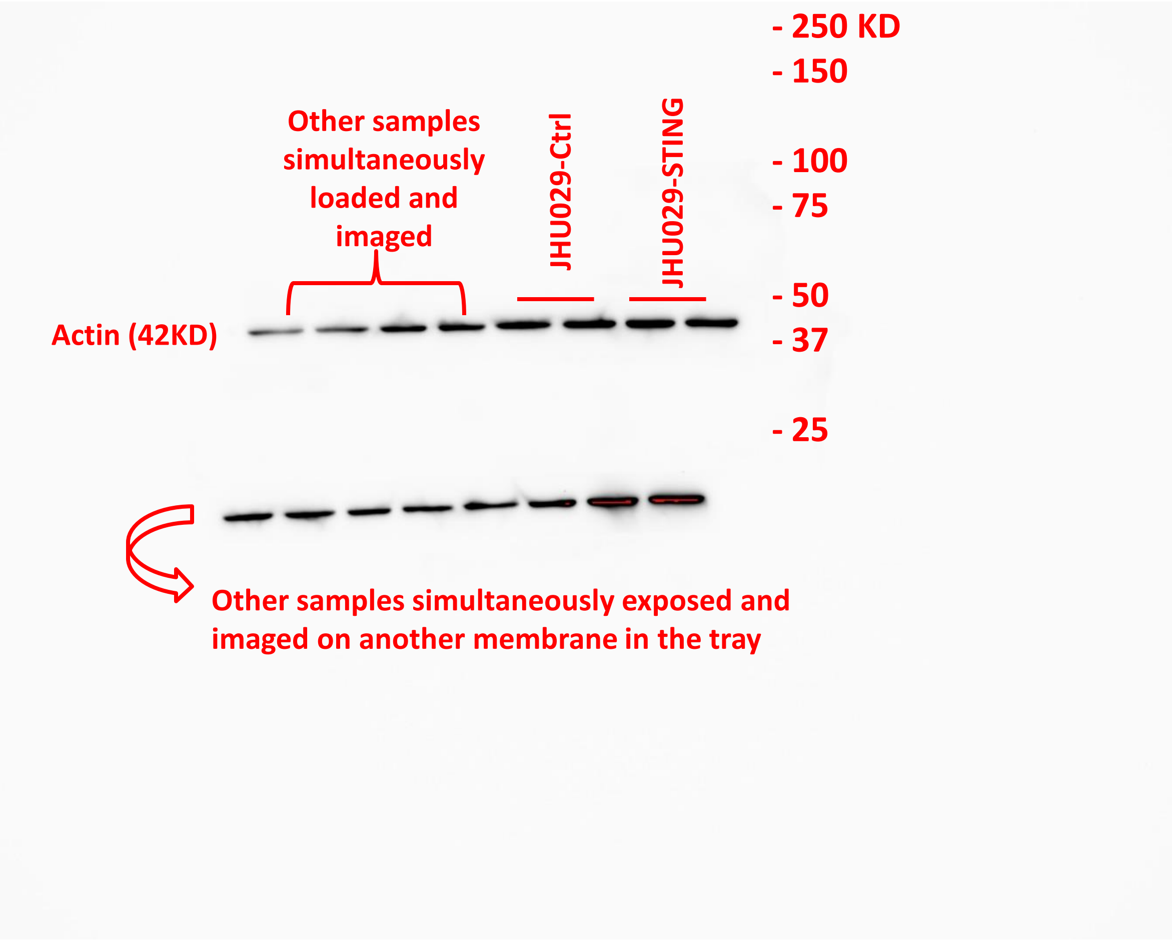

Supplementary Figure 1A. *EGFR* gene expression

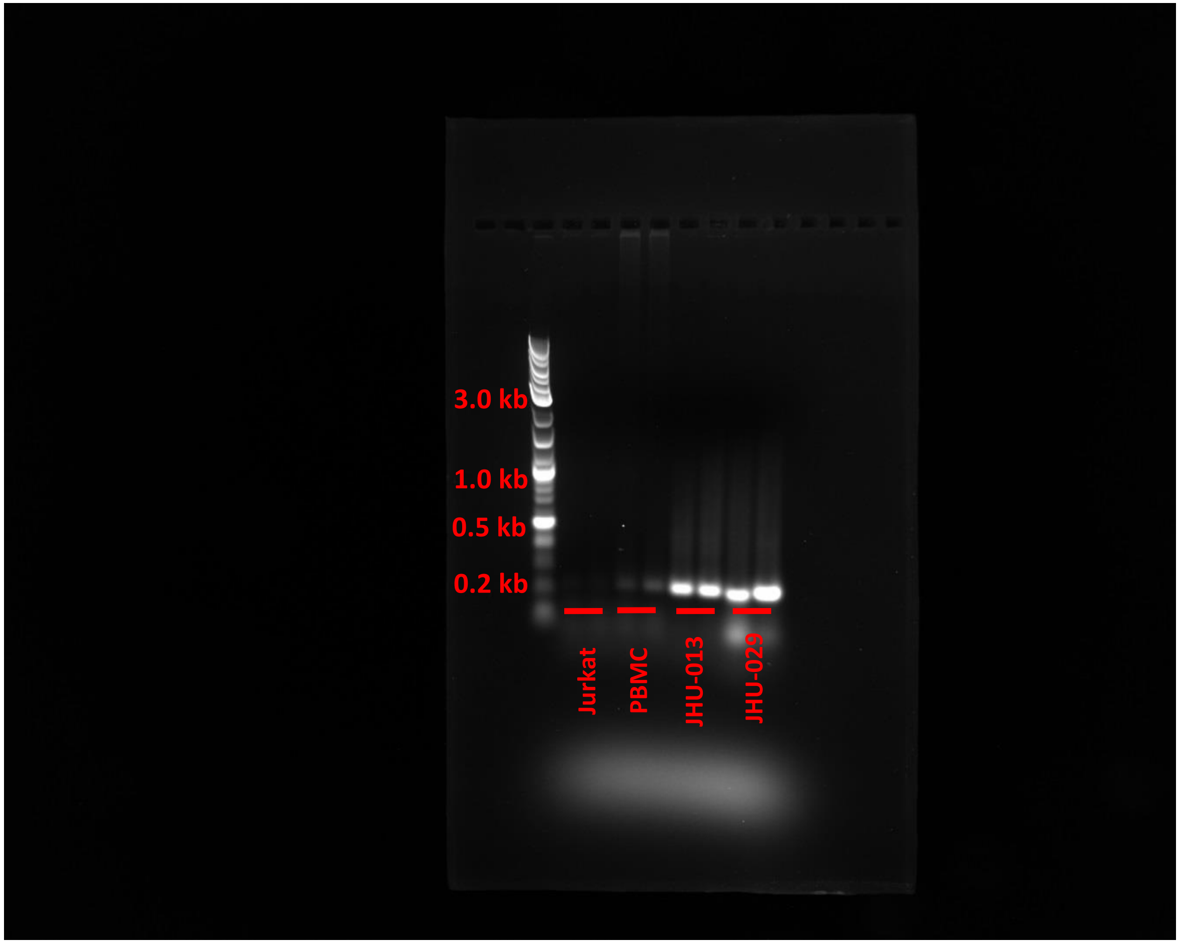

Supplementary Figure 1B. *GAPDH* gene expression

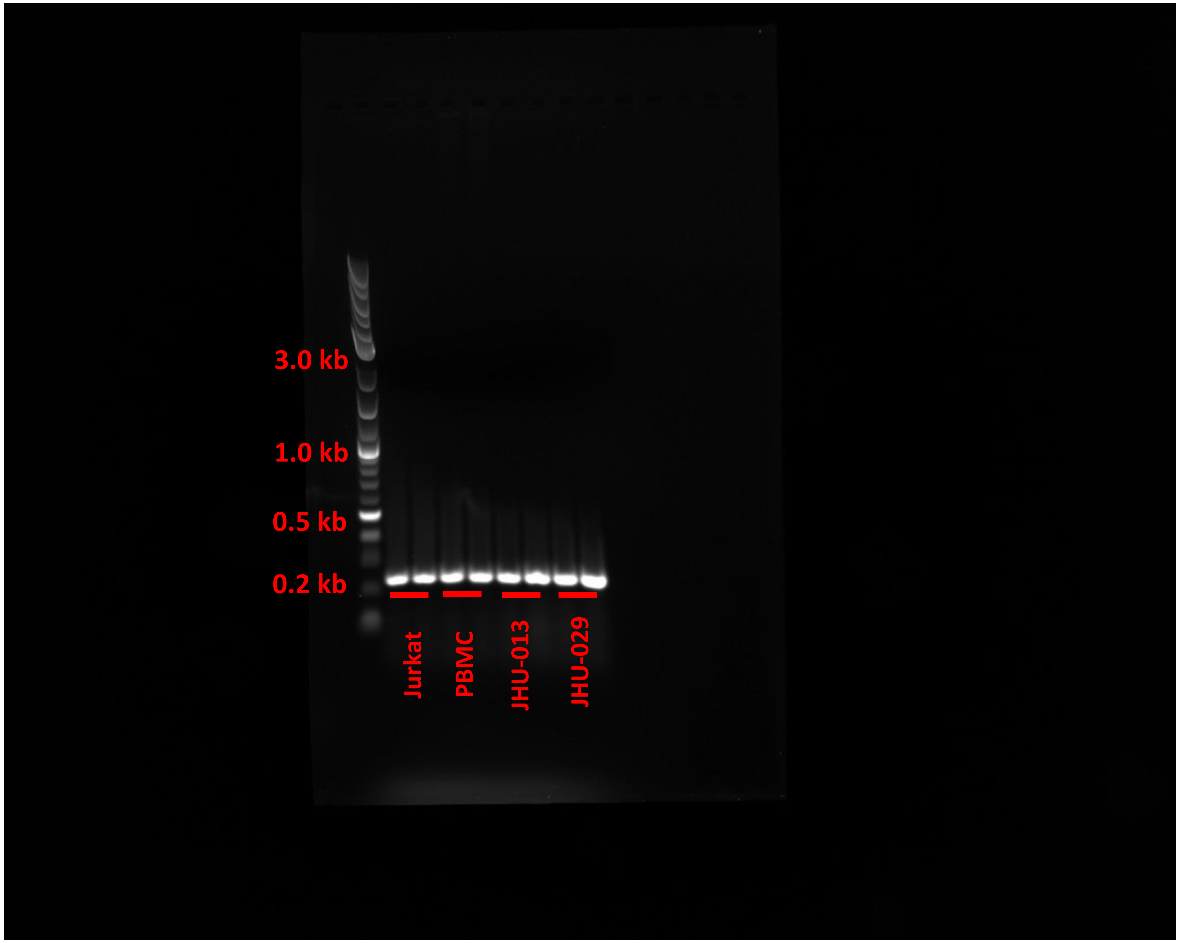

Supplementary Figure 2Aa. STING in JHU013

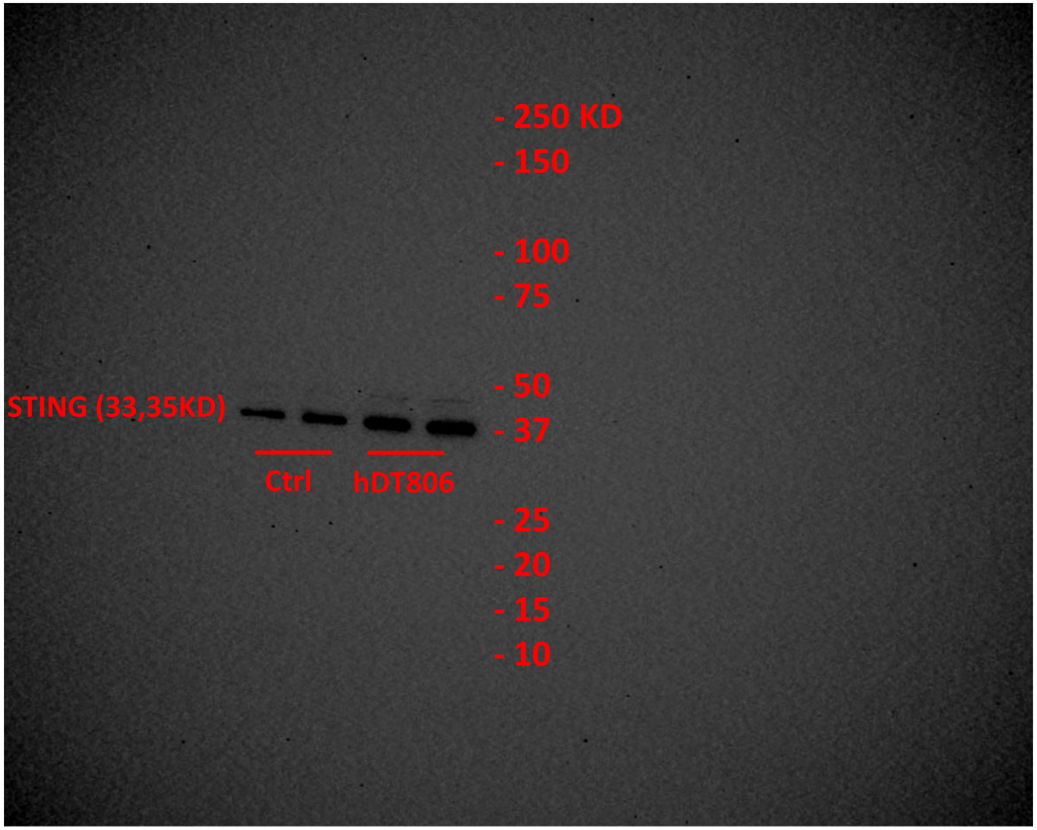

Supplementary Figure 2Ab. pTBK1 in JHU013

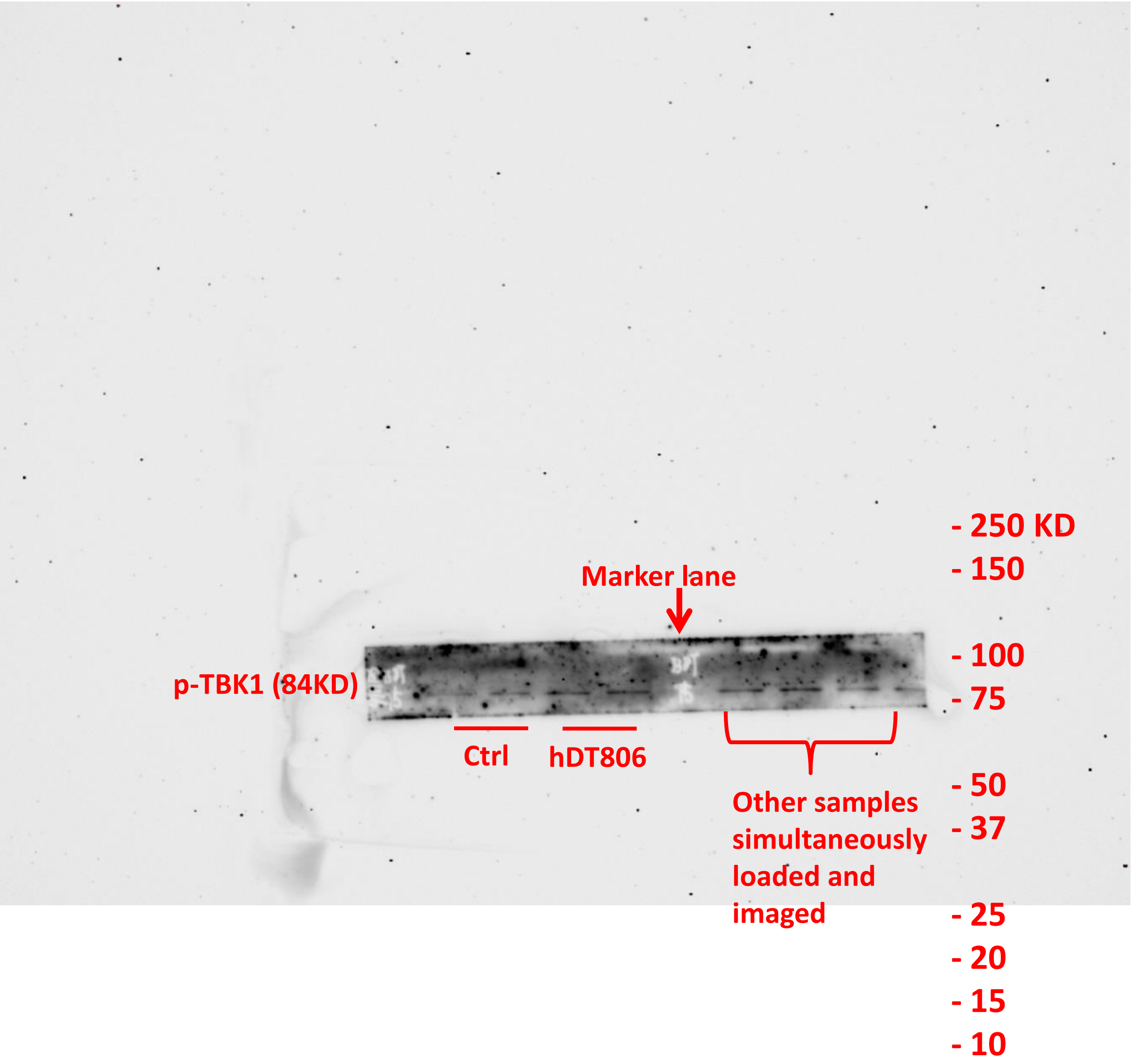

Supplementary Figure 2Ac. TBK1 in JHU013

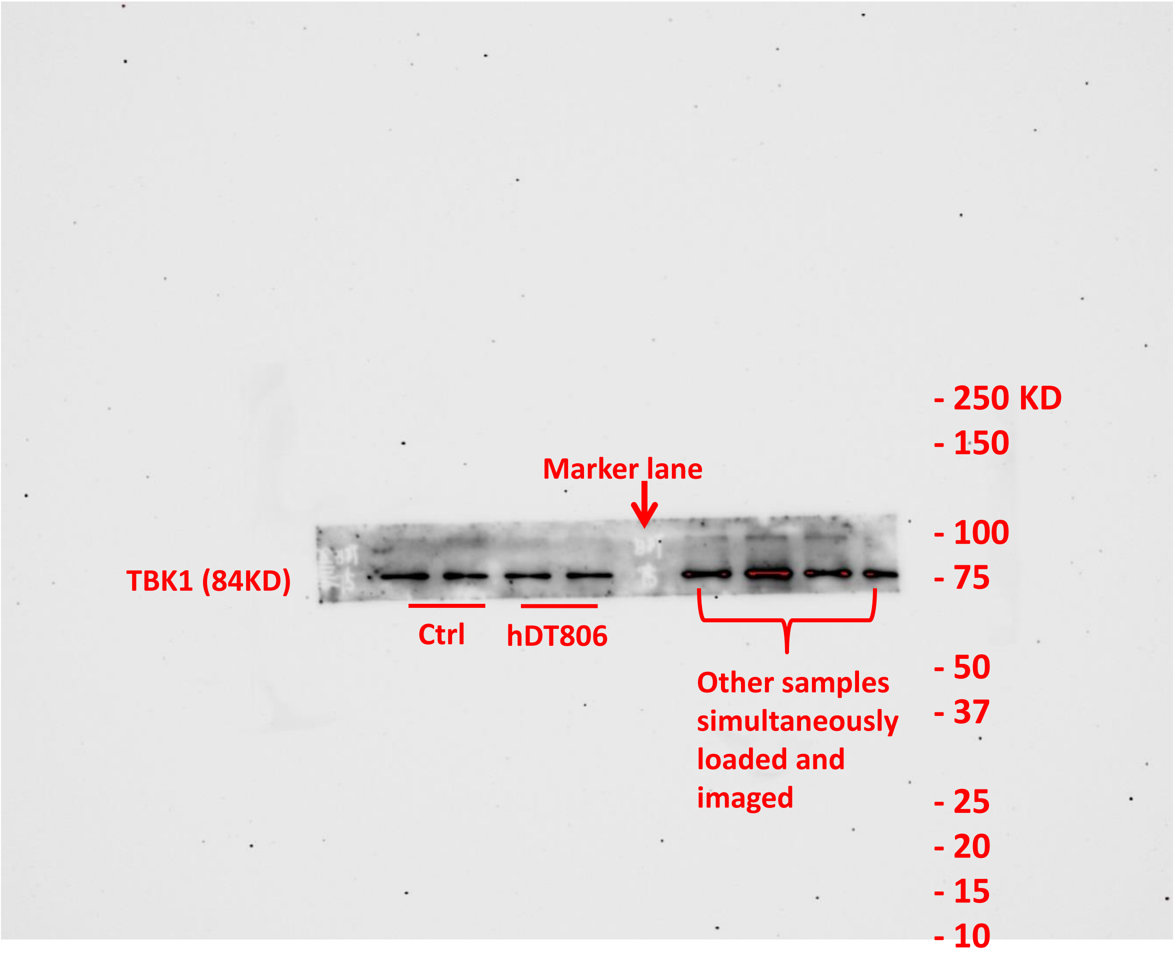

Supplementary Figure 2Ad. MX1 in JHU013

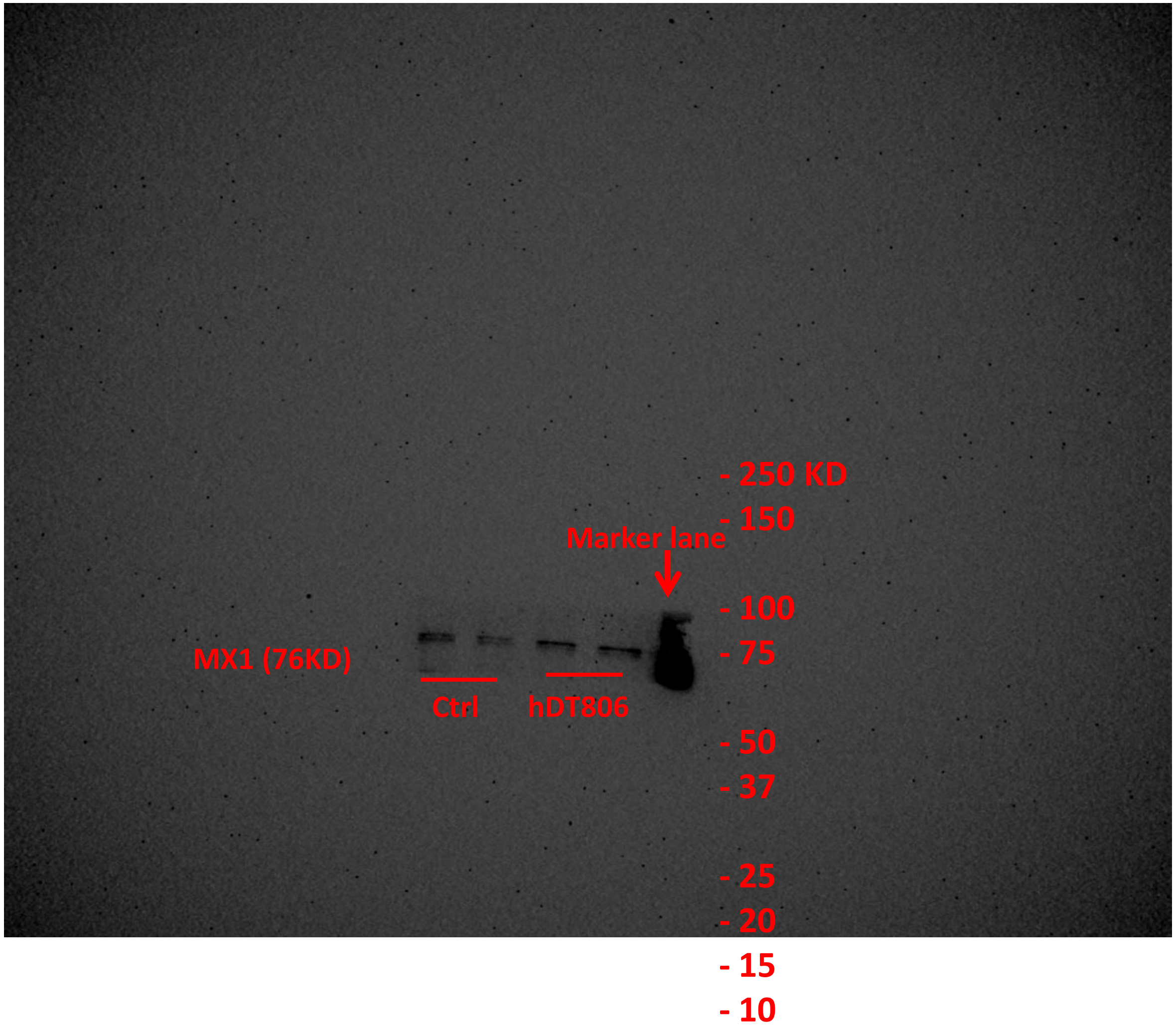

Supplementary Figure 2Ae. Actin in JHU013

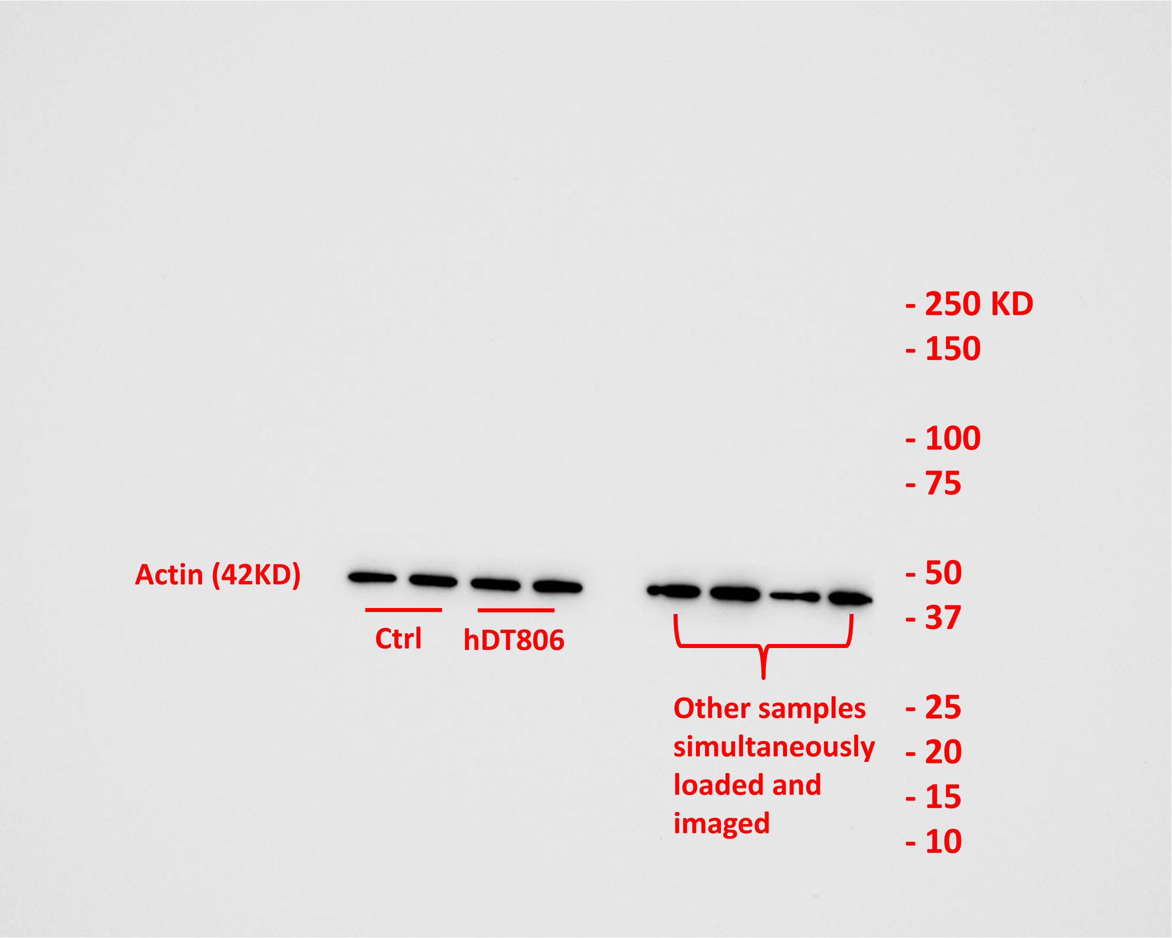

Supplement: Supplementary file 1 — Supplementary Figures. [file 41598_2023_45797_MOESM1_ESM.pdf]
